# State-of-the-art in Metastatic Uveal Melanoma Treatment: A 2025 Update: How to treat Metastatic Uveal Melanoma in 2025

**DOI:** 10.1007/s11912-025-01684-0

**Published:** 2025-05-17

**Authors:** Dimitrios C. Ziogas, Dimitra Foteinou, Charalampos Theocharopoulos, Anastasios Martinos, Dioni-Pinelopi Petsiou, Amalia Anastasopoulou, Helen Gogas

**Affiliations:** First Department of Medicine, School of Medicine, National and Kapodistrian University of Athens, Athens, Greece

**Keywords:** Uveal melanoma, Immune checkpoint inhibitors, Tebentafusp, Immunotherapy resistance, Targeted therapies

## Abstract

**Purpose of review:**

Uveal melanoma (UM) is the most common intraocular malignancy in adults, representing a rare but aggressive melanoma subtype with a distinct molecular landscape, unique metastatic behavior and limited therapeutic options in the metastatic setting. This review provides an in-depth analysis of the latest evidence on the evolving treatment landscape of metastatic UM.

**Recent findings:**

For liver-only metastatic disease, locoregional therapies provide significant benefit compared to systemic therapies. The recent approval of tebentafusp-tebn, a bispecific gp100 peptide-HLA-directed CD3 T-cell engager, marks a pivotal advancement for HLA-A*02:01-positive patients with unresectable/metastatic UM, demonstrating a clinically significant survival benefit. Several clinical studies are currently active, examining emerging locoregional and systemic treatments for metastatic UM, with promising early data.

**Summary:**

Despite effective local disease control through radiotherapy and enucleation, approximately 50% of patients develop metastatic disease, predominantly in the liver, with a median survival of less than one year. The approval of tebentafusp represents a landmark achievement in UM treatment, while promising experimental combinations have demonstrated clinical utility in late phase clinical trials, offering hope for further improvement in patient survival.

## Introduction

Although uveal melanoma (UM) is a relatively rare disease, it is the most common intraocular malignancy in adults, accounting for 83% of all ocular melanomas [1]. UM originates from the extracutaneous melanocytes within the uveal tract of the eye, which comprises the pigmented tissue of the iris, ciliary body and choroid. Approximately 90% of these tumors are located in the choroid, while 6% occur in the ciliary body and 4% in the iris [2]. UM most commonly presents between 50 and 70 years of age and is typically unilateral. Despite sharing the same cellular origin with cutaneous melanoma (CM), UM is a biologically and clinically distinct entity, with a markedly different pathogenesis, mutation spectrum and metastatic progression pattern. With the exception of iris melanoma, UM is not associated to ultraviolet light exposure and is primarily driven by oncogenic mutations in *GNAQ* and *GNA11* compared to *BRAF* and *NRAS* mutations seen in CM [3].

UM is often diagnosed incidentally, with approximately 30% of patients being diagnosed during routine ophthalmologic examination. At the time of diagnosis, over 95% of patients have disease confined to the eye [4]. In such cases, effective local therapeutic options include radiotherapy and enucleation. Despite optimal primary treatment, nearly 50% of UM patients will eventually develop metastatic disease via hematogenous spread, underscoring the challenge of systemic disease control [5]. Disease recurrence typically occurs within the first five years following local treatment and is an ominous predictor of survival. In a recent meta-analysis of 912 patients with metastatic UM, the median progression free survival (mPFS) was 3.3 months, the median overall survival (mOS) was 10.2 months and the 1-year OS rate was 43% [6]. In the majority of patients, distant metastases involve the liver (93%), lung (24%) and bone (16%) [7].

Unlike CM, which has seen remarkable success with targeted therapies and immune checkpoint blockade, these modalities have generally failed to produce clinically meaningful responses in UM. Tebetafusp-tebn is a breakthrough agent that achieved significant survival benefit in comparison with investigator’s choice of therapy in a randomized controll trial (NCT03070392), becoming the first systemic agent to receive regulatory approval for UM. This milestone has revitalized research efforts, driving interest in emerging therapeutic strategies aimed at improving outcomes for localized and metastatic disease. In the present narrative review, we provide a comprehensive overview of the current treatment landscape of metastatic UM with emphasis on recent advancements in locoregional and systematic therapies.

## Genetics

The tumor mutational burden (TMB) quantifies the number of nonsynonymous somatic mutations per genome megabase (Mb) harbored by cancer cells [8]. These mutations are phenotypically manifested by the expression of melanoma-associated antigens (MAAs) that mark melanoma’s immunogenicity, therefore qualifying TMB as an index of melanoma neoantigen burden [8]. MAAs drive T-cell priming, activate, and amplify an effective tumor-specific immune response [9]. High TMB (TMB-H), defined as ≥ 10 muts/Mb, has been validated as a predictive biomarker of immunotherapy response in tumors whose neoantigen load is positively correlated with CD8 + T-cell infiltration [10]. In general, CM bears a high mutational load (median TMB 13.5 mutations/Mb [11]), as a result of the mutagenic impact of ultraviolet radiation [12]. To the contrary, the uveal tract belongs to sun protected areas, which are associated with a low TMB (median of 4 muts/Mb) [13], and exhibits a TMB-L status with a median of 3.2 muts/Mb [14]. Furthermore, genes that are known to predispose to CM development, such as *CDKN2 A* and *CDK4* are not associated with UM [15], with the exception of BRCA1 associated protein-1 (*BAP1*), which is associated with familial cases of UM [16, 17]. Somatic mutations of *GNAQ* and *GNA11 A*, that encode G-protein alpha subunits, are considered to be an early event in the development of UM. Mutations in cysteinyl leukotriene receptor 2 (*CYSLTR2*) and phospholipase C-β4 (*PLCβ4*), located both upstream and downstream of the GNAQ and GNA11 pathway, have been found to occur early in tumorigenesis [18]. *BAP1* is a tumor suppressor gene which encodes a ubiquitin carboxyl terminal hydrolase with deubiquitinase activity, involved in the regulation of different cell functions, including DNA damage repair, cell cycle control, chromatin modification, programmed cell death, and immune response [19]. It has been proposed that all UM patients should undergo immunohistochemical screening for BAP1, as BAP1 mutations are believed to play an important role in metastatic progression and are strongly associated with trisomy of chromosome 3 (trisomy 3) and more aggressive disease [20]. Tumors with disomy 3 exhibit lower metastatic potential and are linked to alterations in the *EIF1 AX*, and *SF3B1* genes [21]. *SF3B1* encodes a component of the splicesome and its mutations lead to alternative splicing while being associated with a relatively favorable prognosis [22]. UM harboring *SF3B1* mutations exhibits unique characteristics, including a delayed onset of metastases, higher propensity for extrahepatic metastatic involvement and prolonged OS [23]. Mobuchon et al. reported a significant association between UM risk and single-nucleotide polymorphisms (SNPs) in pigmentation-related genes, including *IRF4* on chromosome 6, which is related to the disomy 3 UM subtype, and *HERC2* on chromosome 15, which is related to the more aggressive monosomy 3 UM subtype [24].

## Prognosis

### Cytogenetics and transcriptomics

Compelling evidence has associated certain recurrent DNA copy number alterations with the risk of disease progression and metastatic dissemination. Monosomy 3 is an indicator of high-risk melanoma and is associated with worse outcomes in multivariate analysis (HR: 3.19; 95%CI: 1.27–8.02, P = 0.014) [25]. Patients with monosomy 3 tumors experienced a significantly lower 3-year OS and RFS rate compared to patients without monosomy 3 (58% vs. 100% and 50% vs. 100%, respectively) [26]. Furthermore, chromosome 8p loss (HR: 1.97; 95%CI: 1.03–3.77, P = 0.04) and 8q gain are also associated with a grave prognosis, although the latter was marginally significant (HR: 2.30; 95%CI: 0.99–5.30, P = 0.052) [25]. Interestingly, 8q-gain frequently coexists with monosomy 3 and their concomitant presence is associated with worse survival compared to either aberration alone [27]. Chromosome 6p gain is considered mutually exclusive with monosomy 3 and is a predictor of favorable prognosis, while loss of 6q is a negative prognosticator [28]. Lastly, the loss of 1p also appears to play a role in disease progression (HR: 2.16; 95%CI: 1.14–4.06, P = 0.018) [25].

Gene expression profiling (GEP) is another molecular prognostic method used to predict prognosis. In their landmark publication, Onken et al. observed that primary UM cluster into two discrete molecular classes, class 1 (low-grade) and class 2 (high-grade) [29]. The majority of class 1 tumors are associated with disomy 3, 6p gain and are considered low-risk, whereas class 2 tumors are associated with monosomy 3 and other adverse prognostic factoris, including increased age, increased tumor diameter and epithelioid type [29, 30]. In a prospective, multicenter study, GEP emerged as the strongest independent predictor of metastatic dissemination. Importantly, chromosome 3 status did not offer additional prognostic information, independent of GEP, in multivariate analysis [30].

### Histopathology

Various histological factors can affect the prognosis of UM. In a large study of 8033 eyes, Shields et al. reported a significant association between primary tumor thickness and metastatic progression per multivariate analysis (HR: 1.06, 95%CI: 1.03–1.09). For all UM subtypes combined, the rate of metastasis per millimiter increment was 6% for less than 1.0 mm of thickness and reached 51% for tumors thicker than 10.0 mm. [2]. Further independent risk factors for UM metastasis were posterior uvea location (HR: 2.30, 95%CI: 1.10–4.80), tumor diameter (HR: 1.14, 95%CI: 1.11–1.16), tumor pigmentation (HR: 1.41, 95%CI: 1.15–1.73), as well as the presence of subretinal fluid (HR: 1.28, 95%CI: 1.09–1.51), extraocular extension (HR: 1.41, 95%CI: 1.02–1.95) and intraocular hemorrhage (HR: 1.22, 95%CI: 1.01–1.47) [2]. Further unfavorable prognostic factors include epithelioid cell type and a high number of mitoses [31].

### Circulating tumor DNA

Circulating tumor DNA (ctDNA) has emerged as a promising biomarker for predicting and monitoring treatment response in cancer, offering real-time insights into tumor dynamics. By detecting residual disease, serving as a proxy to the disease burden and identifying emerging resistance mutations, ctDNA analysis enables a more personalized and adaptive approach to cancer treatment. In mUM, pretreatment ctDNA is detected in 77 to 84% of patients [32, 33] and is signficiantly associated with disease burden and overall survival. Patients treated with tebentafusp who had detectable ctDNA before treatment commencement had significant lower mOS and mPFS compared to patients with undetectable levels (12.9 months vs. 40.5 months, *P* = 0.001 and 2.5 months vs. 10.8 months, P = 0.001, respectively) [34]. Furthermore, patients with early reduction in ctDNA (90% or greater at 12 weeks) also experienced prolonged mOS compared to patients with lower reduction (21.2 months versus 12.9 months, respectively, *P* = 0.02).

### Prognostic tools

Although the American Joint Committee on Cancer (AJCC) TNM staging system has been widely used as a prognostic tool, this classification does not consider tumor histology or genetic determinants. Enhancing the AJCC staging with chromosome 3 and 8q status or the tumor gene expression profile could provide a more comprehensive prognostic estimate [35]. The Cancer Genome Atlas Program (TCGA) prognostic groups A to D were organized based on chromosome 3 and 8q copy numbers, creating four clusters, that include Group A (disomy 3, disomy 8), Group B (disomy 3, 8q gain), Group C (monosomy 3, 8q gain possible), and Group D (monosomy 3, 8q gain multiple), with 5-year rate of metastasis calculated at 4%, 12%, 33% and 60%, respectively [36]. Furthermore, the Liverpool Uveal Melanoma Prognosticator Online (LUMPO) is a useful, as well as widely available tool that utilizes known clinical, histological, and genetic determinants, along with age and sex to evaluate both metastatic and non-metastatic mortality [37]. A similar tool for patients that receive treatment for primary UM is PriMeUM. This tool is used to predict metastases within 48 months of treatment of a primary uveal melanoma [38]. Additionally, a number of DNA sequencing methods are able to identify *EIFA1X, SF3B1* and *BAP1* mutations. These are available both commercially, with the most notable being the DecisionDX-UMSeq, and as institutional – specific assays [39]. DecisionDX-UMSeq is a 15-gene expression profile test, which subdivides UMs into class 1 A low-risk tumors, class 1B intermediate-risk tumors and class 2 high-risk tumors. Finally, preferentially expressed antigen in melanoma (PRAME) serves as an independent biomarker capable of identifying higher-risk subgroups among patients with Class 1 or disomy 3 tumors [40]. The Collaborative Ocular Oncology Group Study Number 2 (COOG2) described superior prognostic accuracy of combining a 15-gene expression profile (15-GEP) and PRAME when compared to 15-GEP alone. The most important independent predictor of MFS was 15-GEP (HR: 5.9; 95% CI, 4.43 to 7.99), followed by PRAME status (HR: 1.82; 95% CI, 1.42 to 2.33) [41]. Moreover, elevated PRAME expression was found to be related with earlier metastasis and melanoma-specific mortality in patients with DecisionDX-UM class 2 tumors [42].

## Treatment of metastatic disease

**Fig. 1:**
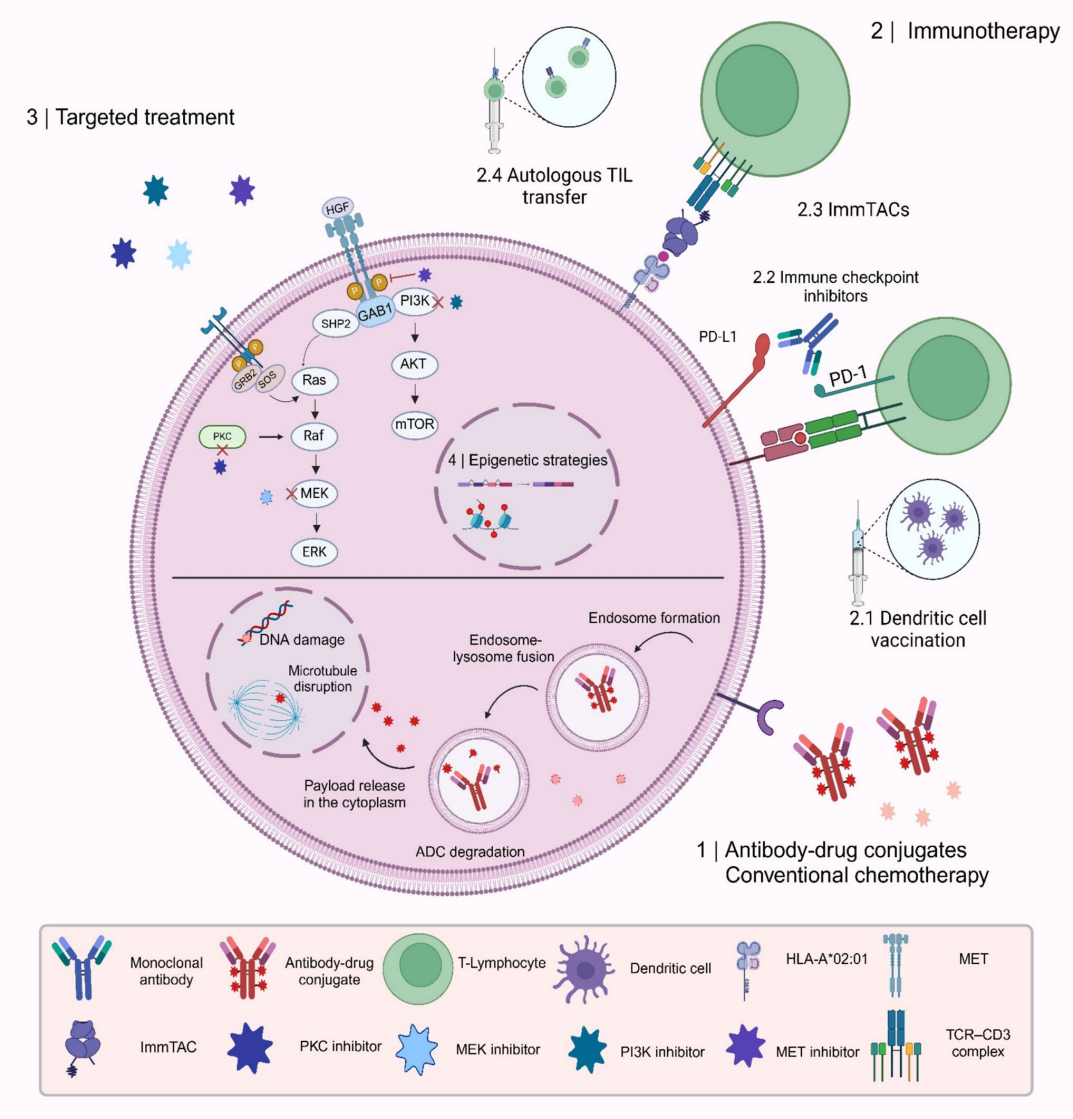
Mechanism of action of systemic treatment modalities for metastatic uveal melanoma. TIL: tumor infiltrating lymphocytes; ImmTAC: Immune-mobilizing monoclonal T cell receptors Against Cancer; ADC: antibody–drug conjugate

The therapeutic landscape of mUm continues to evolve, with patient-specific factors playing a pivotal role in guiding therapeutic planning. Key considerations include the performance status, HLA-A*02:01 genotype testing, tumor burden and growth kinetics as well as the accessibility of relevant clinical trial. Many systemic therapies that have demonstrated efficacy in CM have been explored in UM, including ICIs and molecularly targeted treatments. However, these approaches have generally produced modest, at best, clinical benefit in UM, fueling significant research interest for the development of effective strategies. In cases of liver-dominant disease, locoregional strategies have shown promising results. However, in patients with extrahepatic dissemination, systemic treatment modalities are generally favored to address the multifocal nature of metastatic progression. Figure 1 demonstrates the mechanism of action of systemic therapies. For treatment-naïve patients with metastatic disease who test positive for HLA-A*02:01, tebentafusp – a bispecific T-cell receptor fusion protein – has received regulatory approval and is currently a first-line therapy. In contrast, for HLA-A*02:01-negative patients, enrollment to relevant clinical trials is suggested. Table 1 summarizes recruiting clinical trials in mUM. Of note, however, early data from the combination of Darovasertib and crizotinib suggest superior survival benefit compared to current standards of care and could potentially lead to an accelerated approval for this patient population.

**Table 1:** Summary of active, recruiting clinical trials investigating locoregional or systemic treatments for metastatic uveal melanoma

Clinical trial identifier	Level of testing	Line	Intervention	Types of drugs
NCT06519266	III	NS	PHP plus ipilimumab and nivolumab versus ipilimumab and nivolumab	Ipilimumab: anti-CTLA4 mAb Nivolumab: anti-PD-1 mAb
NCT06581406	II/III	NS	RP2 plus nivolumab versus ipilimumab plus nivolumab	RP2: Oncolytic virus Nivolumab: anti-PD-1 mAb Ipilimumab : anti-CTL4 mab
NCT05987332	II/III	1L	Crizotinib plus darovasertib	Crizotinib : MET/ALK multi-targeted TKIDarovasertib : Protein kinace C inhibitor
NCT05170334	II	NS	Binimetinib plus belinostat	Binimetinib: MEK inhibitor
NCT06121180	II	NS	Cemiplimab plus afibercept	Cemiplimab: anti-PD-1 mAb Afibercept: VEGF inhibitor
NCT06627244	II	NS	SIRT plus tebentafusp	Tebentafusp: gp100 peptide-HLA-directed CD3 T cell engager.
NCT03467516	II	NS	Adoptive TIL transfer plus high-dose aldesleukin	Aldesleukin: recombinant IL-2
NCT05315258	II	1L	Single-agent tebentafusp	Tebentafusp: gp100 peptide-HLA-directed CD3 T cell engager.
NCT06070012	II	1L	Single-agent tebentafusp	Tebentafusp: gp100 peptide-HLA-directed CD3 T cell engager.
NCT06717126	II	>2L	Single-agent roginolisib versus BAC	Roginolisib:PI3 Kδ allosteric modulator
NCT05077280	II	NS	SBRT plus relatlimab and nivolumab	Relatlimab: anti-LAG-3 mAbNivoluman: anti-PD-1 mAb
NCT05524935	II	NS	Olaparib plus pembrolizumab	Olaparib: poly(ADP‐ribose) polymerase inhibitorPembrolizumab: anti-PD-1 mAb
NCT06703398	II	>2L	TIL versus investigator’s choice of chemotherapy	N/A
NCT05415072	I/II	NS	Single-agent DYP688	DYP688: anti-PMEL17 ADC
NCT06626516	I/II	1L	LDT plus tebentafusp	Tebentafusp: gp100 peptide-HLA-directed CD3 T cell engager.
NCT03947385	I/II	NS	Darovasertib alone or in combination with binimetinib or crizotinib	Darovasertib: Protein kinace C inhibitorBinimetinib:MEK inhibitor Crizotinib: MET/ALK multi-targeted TKI
NCT03611868	I/II	NS	Single-agent APG-115 versus APG-115 plus pembrolizumab	APG-115 : MDM2 inhibitorPembrolizumab : Anti-PD-1 mAb
NCT05496686	I	>2L	Single-agent 225 Ac-MTI-201	225 Ac-MTI-201: Targeted alpha particle radiotherapy
NCT05607095	I	NS	Adoptive TIL	N/A
NCT06805825	I	>2L	Single-agent NN2301	NN2301 :Anti-c-Kit ADC

PHP: percutaneous hepatic perfusion; SIRT: selective internal radiation therapy; LDT: liver-direct treatment; TIL: tumor infiltrating lymphocytes; ADC: antibody-drug conjugates; BAC: best alternative care; mAb: monoclonal antibody; TKI: tyrosine kinase inhibitor

### Locoregional therapies

UM demonstrates strong metastatic organotropism with a very high predilection for hepatic metastases. At the time of death, liver metastases are observed in approximately 90% patients, while in 50% of these cases, metastases are confined only to the liver [7]. Given the hepatic tropism of UM, liver-targeted treatment options hold promise for enhancing patient outcomes. In a large meta-analysis of 912 mUM patients, liver-directed treatment, provided significant survival benefit compared to systemic pharmacotherapy, achieving a mOS of 14.6 versus 9.3 months and a mPFS of 5.2 versus 2.8 months, respectively [6].

#### Surgical treatment

In selected patients with limited metastatic burdern, hepatectomy confers significantly improved long-term survival. In the largest study to date (n = 255 patients), Mariani et al. reported that the mOS following surgery, irrespective of the adequacy of resection, was 14 months compared to 8 months in patients managed nonoperatively. Importantly, microscopically complete resection provided significant survival benefit, achieving a mOS of 27 months and a median recurrence free survival of 19 months [43]. Predictors of OS after surgery per multivariate analysis included the time to liver metastasis (> 24 months), R0 resection, number of liver metastases (< 5) and absence of miliary disease. Several smaller studies have also reported similar outcomes [44–46]. To the contrary, although transplant oncology has produced promising results, liver transplantation for ocular melanoma metastases remains a formidable pursuit. In the only published study to date, Dueland et al. reported two cases of liver m UM metastases treated with liver transplantation[47]. The first patient had a large tumor load, with the largest metastasis measuring 160 mm, and suffered disease recurrence 5.6 months after OLT, while the second patient experienced recurrence at 14.7 months and died 26.6 months after OLT.

#### Ablative treatment

Limited, yet promising, data exist on the usefulness of thermal ablation for UM hepatic metastases using radiofrequency ablation (RFA) and laser-induced thermotherapy (LITT). LITT relies on the precise delivery of light laser energy through an optical fiber inserted into the target tissue, where tissue chromophores convert it into photothermal energy [48]. Radiofrequency ablation (RFA) uses high-frequency alternating current to create an electric field that induces ionic agitation in the target tissue, generating heat. In both techniques, the accumulating heat causes protein denaturation and leads to coagulative necrosis, effectively destroying cancerous tissue, while minimizing harm to surrounding structures. Bale et al. observed notable efficacy with stereotactic RFA in six patients with mUM, reporting a mOS of 38.0 months, which was significantly higher compared to CM patients (11.6 months) [49]. The mDFS was 9.3 months for the totality of the cohort. In another study by Mariani et al., RFA with or without surgery achieved similar survival benefit compared to surgery (mOS of 28 vs. 27 months and mDFS of 7 months vs. 10 months, respectively) [50]. Furthermore, Eichler et al. reported excellent outcomes with MR-guided LITT in 18 mUM patients, with a mOS of 2.8 years and a 5-year survival rate was 17% [51]. The mOS after LITT was 2.8 years and the 5-year survival rate was 17%. However, these outcomes are yet to be corroborated in larger-scale studies.

#### Regional chemotherapy

The rationale behind hepatic intra-arterial chemotherapy (HACT) relies on the predominantly arterial vascularization of liver metastases in comparison to the dual, portal-predominant, blood supply of the hepatic parenchyma [52]. This allows for a higher concentration of the administered chemotherapeutic at the tumor site with lower systemic toxicity. HACT is most commonly performed via a subcutaneous pump connected to an intra-arterial catheter inserted into the gastroduodenal artery. Intra-arterial chemotherapy for liver UM metastases can be delivered using three methods, namely hepatic artery infusion (HAI), isolated hepatic perfusion (IHP) and percutaneous hepatic perfusion (PHP).

In HAI, the administered drug circulates throught the liver but is not isolated from systemic circulation. Several studies have investigated HAI using fotemustine, carboplatin, IFN-a plus IL-2 or cisplatin plus vinblastin and dacarbazine in UMLM, reporting a mOS ranging from 11.5 to 16.0 months [53, 54]. In the largest study to date (NCT00110123), a total of 171 patients were randomized to receive either IV or HIA fotemustine. HIA treatment was associated with an improved ORR and 1-year PFS rate (10.5% vs. 2.4% and 24% vs. 8%, respectively), which however did not translate into a survival benefit (mOS of 14.6 vs. 13.8 months, HR:1.09; 95%CI: 0.79–1.50) [55].

IHP involves vascular exclusion of the liver from the systemic circulation via establishment of an external perfusion circuit, via catheter insertion into the hepatic artery and veins. In the phase III SCANDIUM trial (NCT01785316), 93 patients were randomized to either IHP with melphalan or investigators choice of treatment; IHP demonstrated significantly better outcomes, achieving an ORR of 40% versus 4.5%, a mOS of 21.7 versus 17.6 months (HR: 0.64; 95%CI: 0.37–1.10) and a mPFS of 7.4 compared to 3.3 months (HR = 0.21, 95% CI, 0.12 to 0.36) in the control group, respectively [56].

A non-surgical alternative to IHP is PHP, which involves a veno-venous bypass, via occlusion of the inferior vena cava with a double-balloon catheter, which redirects the outflow of blood from the hepatic veins into a filter system, which clears melphalan and subsequently returns blood through a jugular vein sheath. In the phase III FOCUS trial (NCT02678572), eligible patients with UM metastatic to the liver with or without limited extrahepatic disease were randomized to eiterh PHP with melphalan or investigator’s choice of TACE, pembrolizumab, ipilimumab or dacarbazine. Although the study began as a randomized trial, reqruitment in the control group was eventually halted after enrollment of 32 patients and it was subsequently continued as a single-arm study. PHP demonstrated clinically meaningful activity, conferring an ORR of 36.3% (CR in 7.7% and PR in 28.6%), a mOS of 20.5 months and a mPFS of 9.0 months [57]. The efficacy of PHP was consistent in patients with either hepatic-only or extrahepatic disease (ORR of 37.5% vs. 33.3%, respectively). To the contrary, BAC resulted in significantly worse outcomes, with an ORR of 12.5%, DCR of 37.5%, mPFS of 3.12 months and mOS of 14.0 months.

#### Embolization treatment

Several types of embolotherapy have been used in the treatment of UM hepatic metastases. Transarterial chemoembolization (TACE) involves embolization of the hepatic artery with concurrent infusion of chemotherapy. Since it was first attempted by Carrasco et al. [58], TACE has been used for the treatment of UM hepatic metastases for four decades. Several combination of different occlusive agents, primarily polyvinyl alcohol and gelatin sponge, and chemotherapy agents, including cisplatin, mitomycin C, doxorubicin and fotemustine, have been evaluated with wide variation in the published results (ORR ranging from 0 to 57% and mOS of 5.0 months to 23.0 months) [59]. This heterogeneity stems primarily from differences in dosing and study design, given that some authors treated only patients with low hepatic metastatic burden, while others recruited patients with more than 50% metastatic involvement of the liver. To this end, TACE tends to confer greater survival benefit in patients with lower metastatic burden. Sato et al. reported a mOS of 19.0 months in patients with tumor involvement in less than 20% of the liver versus 5.6 months in cases with greater than 20% involvement. The angiographic pattern of the disease is another prognostic factor, with patients with an infiltrative pattern experiencing significantly worse outcomes (mOS of 4.0 months vs. 13.0 months in the nodular group, P < 0.001) [60]. Beyond conventional TACE, drug-eluting bead TACE (DEB-TACE) employs permanent embolic microspheres which are chemically attached to chemotherapeutics, leading to high local concentration of chemotherapy and restricted systemic release. DEB-TACE with irinotecan with or without systemic chemotherapy has been tested in comparison with systemic chemotherapy alone, achieving a mOS of 16 versus 12 months, respectively [61]. Immunoembolization with GM-CSF emulsified in lipiodol has also demonstrated promising clinical activity, outperforming both BCNU-TACE and bland transarterial embolization in terms of mOS and mPFS [62, 63]. Lastly, radioembolization using selective internal radiation therapy (SIRT) has also demonstrated positive results in UMLM. SIRT achieves delivery of high-dose radiation to the metastatic site, while reducing collateral damage to adjacent healthy cells. Due to their small size, 90-Y microspheres become lodge in the microarterial circulation. Given that metastatic sites derive their afferent blood supply from the hepatic artery, these microspheres preferentially become trapped in the vascular supply of metastases. SIRT using Yttrium-90-bearing resin micropsheres showed superior clinical activity comparing to TACE (ORR of 5% in both arms, mPFS of 4.9 vs. 2.2 months, median liver-specific PFS of 8.3 vs. 2.2 months, respectively) in an interim analysis of the phase II SirTac study (NCT02936388), warranting further investigation [64].

### Systemic Chemotherapy

#### Conventional chemotherapy

A plethora of studies have been conducted to evaluate traditional chemotherapy schemes in the treatment of advanced UM. Single-agent regimens that have been evaluated in prospective clinical trials include dacarbazine, paclitaxel, temozolomide, fotemustine, bendamustine, vincristine, lenalidomide and treosulfan [65–68]. Monotherapy with any of these agents resulted in very low ORRs and poor survival benift, with a mPFS lower than 4 months and a mOS of approximately 10 months. A chemotherapy triplet comprising cisplatin, dacarbazine and vinblastine achieved slightly better outcomes with an ORR of 20%, a mOS of 13 months and a mPFS of 5.5 months [69].

#### Antibody–Drug Conjugates (ADCs)

ADCs are essentially tripartite prodrugs comprising a mAb, targeting a tumor-specific antigen, tethered to super-toxic payload via a chemical linker [70]. Their design harness the intricate preferential binding of mAbs, which act as scaffold carriers for the attached chemotherapeutics, selectively targeting tumor cells and sparring adjacent tissues, leading to an enhanced therapeutic index. To date, 16 ADCs haver received regulatory approval as early line of therapy and many more are being tested in late-stage clinical trials, demonstrating promising clinical efficacy even for difficult-to-treat malignancies [71, 72]. DEDN6526 A, is a humanized IgG1 mAb targeting the endothelin B receptor conjugated to the microtubule disrupting agent, MMAE. This ADC was tested in a phase I trial in 53 patients with metastatic or unresectable melanoma including 3 with UM, and produced PRs in six patients (11%) and stable disease lasting more than six months in 17 patients (32%), including all UM patients [73]. Glembatumumab vedotin (GV) consists of a fully human IgG2 anti-glycoprotein NMB mAb linked to MMAE. GV was evaluated in a phase II study in 35 patients with metastatic UM and yielded PRs in two patients (6%) and SD in 51% and achieved a mOS of 11.9 months and a mPFS of 3.1 months [74]. NN3201 is an c-kit-targeted ADC, comprising a fully human mAb conjugated to MMAE, which is currently under clinical investigation in a phase I trial (NCT06805825) for solid tumors known to express c-Kit. DYP688 is a first-in-class ADC comprising an anti-PMEL17 mAb tethered to the Gq/11 inhibitor, SDZ475, which is tested in a phase I/II study (NCT05415072) for patients with metastatic UM harboring GNAQ/11 mutations*. *In vivo*,* DYP688 exerted durable tumor regression in GNAQ/11-mutant UM models, including patient-derived xenografts and liver metastasis models [75].

### Immunotherapy

#### Immune checkpoint inhibitors

In sharp contrast to CM, single-agent CTLA-4 or PD-1 inhibition is associated with very limited clinical activity in patients with metastatic UM, owing to primary or acquired resistance mechanisms [76]. Ipilimumab monotherapy was tested in a phase II, single-arm study (NCT01355120) in pretreated or treatment naïve mUM patients, yielding disappointing results with an 1-year and 2-year OS rate of 22% and 7%, respectively and a mOS of 6.8 months [77, 78]. Similarly, ipilimumab plus liver radiofrequency ablation did not produce any ORs and resulted in a mOS of 9.7 months [79]. Nivolumab was evaluated as second-line treatment in 25 patients after initial treatment with ipilimumab, which had produced SD in 36%, and achieved a mPFS of 3 months and a 1-year surival rate of 28% [80]. First-line nivolumab achieved a PR in 11.7% and SD in 35.3, conferring a mPFS of 3.8 months for the overall population and a mPFS and mOS of 9.7 and 12.8 months, respectively, for patients with an interval higher than 5 years from primary diagnosis [81]. Lastly, single-agent tremelimumab was evaluated in ICI-naïve mUM patients in a multicenter phase II study, which was eventually terminated due to futility at the first interim analysis. Based on an analysis of the eleven enrolled patients, tremelimumab achieved no ORs and resulted in a mPFS of 2.9 months and a mOS of 12.8 months [82].

To the contrary, dual IC blockade has produced more promising outcomes. GEM-1402, a phase II trial (NCT02626962), evaluated nivolumab plus ipilimumab in 52 patients with previously untreated mUM and produced positive results, yielding a 1-year OS of 51.9%(95% CI, 38.3 to 65.5) and a median OS of 12.7 months (95% CI, 7.1 to 18.3), a mPFS of 3 months and an overall DCR of 63.5%, with manageable toxicity [83]. Another phase II trial (CA184-187, NCT01585194) demonstrated similar results upon ipilimumab plus nivolumab coadministration, with an ORR of 18%, a 1-year OS of 56%(95% CI, 38% to 71%) and a median OS of 19.1 months (95% CI, 9.6 months to NR) [84]. With regard to safety, 40% of patients experienced grade 3 and 4 immune-mediated adverse events. The clinical utility of nivolumab plus ipilimumab was further consolidated in larger, multicenter retrospective studies, reporting an ORR ranging from 11.1% to 15.6% and a mOS of 15.0 to 16.1 months [85, 86]. This ICI doublet is currently under investigation in combination with percutaneous hepatic perfusion with melphalan in a phase I/II trial (NCT04283890) and a TLR9 agonist, SD-101, via pressure-enabled drug delivery in a phase I study (NCT04935229, PERIO-01). According to a published preliminary analysis of seven patients from the former study, one patient experience a CR, five had PRs and one had stable disease, with 3 ongoing responses after a median follow-up of 29.1 months [87]. Similarly, early data from the PERIO-01 trial suggest promising biological activity [88]. Nivolumab was also tested in combination with relatlimab in a phase II study (NCT04552223), confering a low response rate (ORR of 7.7%) in the 27 enrolled patients [89]. Nivolumab plus relatlimab is currently under investigation with concurrent stereotactic body radiotherapy for mUM in a phase 2 trial (NCT05077280).

Furthermore, ICIs are also being evaluated in combination with other systemic pharmacotherapies. LNS8801, an oral G protein-coupled estrogen receptor-1 (GPER) agonist is currently being tested in a phase I/II study (NCT04130516) both as a monotherapy and in combination with pembrolizumab for treating solid tumors, including UM. In melanoma mice models, activation of GPER signaling led to inhibition of melanoma cell proliferation, heightened melanocytic differentiation and augmented response to PD-1 inhibition [90]. In a preliminary analysis of 14 heavily pretreated UM patients from the NCT04130516 study, LNS8801 achieved disease control in seven patients (4 as monotherapy and 3 in combination with pembrolizumab), including one with a PR [91]. Importantly, germline GPER was present in only two of the 12 sequenced patients, indicating a significant over-representation of the hypofunctional GPER variant comparing to the general population. This potentially suggest even better outcomes in carefully selected patients with concensus germline GPER. Enhanced response to anti-PD-L1 treatment has also been observed upon concurrent mouse double minute 2 homolog (MDM2) antagonism. MDM2 is an oncoprotein that binds the p53 tumor suppressor protein and promotes ubiquitin-mediated degradation and p53 nuclear export, inhibiting its transcriptional activity [92]. APG-115, a MDM2 antagonist, was shown to activate p53 and p21, increase M1 macrophage polarization and achieve significantly enhanced anitumor activity in combination withn PD-1 blockade, compared to PD-1 inhibition alone [93]. These promising preclinical data propelled APG-115 (alrizomadlin) into clinical trial testing for solid tumors including UM. According to preliminary results from an active phase I/II study of APG-115 plus pembrolizumab, the investigational combination achieved an ORR of 9% in the UM subcohort (one PR among 11 patients) [94]. Lastly, ICIs targeting beyond immune checkpoints beyond CTLA-4 and PD-1 exhibit promising antineoplastic effects in mCM, warranting investigation in mUM [95].

#### Tumor vaccines

Limited data exist on the efficacy of therapeutic cancer vaccines in metastatic UM. In one of the most promising studies to date, Bol et al. tested dendritic cell vaccination in 14 patients with metastatic UM. Dendritic cell-vaccinated patients had a mOS of 19.2 months, including a mOS of 29 months for patients with M1a disease [96]. A phase I vaccination trial (NCT04335890) using Inhibitory kappa B kinase beta-matured dendritic cells loaded with autologous tumor RNA and RNA coding for defined antigens and driver mutations in combination with checkpoint blockade is also currently active [97].

#### Oncolytic virotherapy

Oncolytic virotherapy (OV) is an emerging strategy that uses naturally ocurring or genetically engineered viruses to selectively kill tumor cells via a dual mechanism; virus mediated-lysis and the induction of antitumor immune cells [98]. OV has been investigated in mUM with mixed outcomes. RP2 is an enhanced-potency oncolytic herpes simplex virus type 1 which expresses granulocyte–macrophage colony-stimulating factor, fusogenic gibbon ape leukemia virus glycoprotein with the R sequence deleted and an anti–CTLA-4 antibody-like molecule. According to preliminary data from the UM cohort (n = 17) of the phase I NCT04336241 basket study, RP2 as a monotherapy (n = 3) or in combination with nivolumab demonstrated significant antitumor activity in pretreated patients, achieving an ORR of 29.4% and a DCR of 58.8%, while maintaing an excellent safety profile [99]. RP2 is also currently being investigated in a phase II/III clinical trial (RP2-202, NCT06581406) in combination with nivolumab versus ipilimumab plus nivolumab in ICI-naïve mUM patients. On the contrary, single agent vesicular stomatitis virus vector modified to express interferon-beta tyrosinase related protein 1, ICOVIR-5, an oncolytic adenovirus, and combination of V937 (Coxsackievirus A21) with ipilimumab failed to induce meningful clinical benefit [100–102].

#### Autologous tumor-infiltrating lymphocytes

Adoptive transfer of tumor-infiltrating lymphocytes (TILs) involves a multistep process of isolating, expanding and reinfusing autologous T cells with tumor reactive potential. TIL therapy has demonstrated durable responses in patients with advanced CM and offers a promising strategy for overcoming the immunosuppressive UM tumor microenvironment. In the first phase II study (NCT01814046) of adoptive transfer of autologous TILs in mUM, ORs were observed in six out of 20 enrolled patients (35%), including six PRs and one CR [103]. TIL therapy is being further evaluated in a phase II study (NCT00338377) in metastatic melanoma patients, including UM. According to preliminary data from the UM cohort, TIL therapy was feasible in 9 out of 96 enrolled patients who underwent TIL harvest. All patients had liver metastases and had undergone a median of 3 prior lines of treatment. TIL therapy achieved a DCR of 66%, including 22% PRs and 44% SD [104]. TIL therapy is currently being investigated for patients with mUM in a phase I (NCT05607095) and two phase II clinical trials (NCT03467516, NCT06703398).

#### Immune-mobilizing monoclonal T cell receptors Against Cancer (ImmTACs)

Tebentafusp (KIMMTRAK ®) is a first-in-class immune-mobilizing monoclonal T cell receptor Against Cancer (ImmTAC) that targets the melanocyte lineage-specific gp100 peptide, presented exclusively by HLA-A*02:01. It consists of a high-affinity soluble T-cell receptor fused to an anti-CD3 single-chain variable fragment, enabling selective binding to gp-100 expressing melanoma cells, while recruiting and activating T-cells. Tebentafusp was granted FDA and EMA approval as a monotherapy for the treatment of mUM, following the outcomes of the pivotal phase III trial IMCgp100-202 (NCT03070392), which compared single-agent tebentafusp to investigator’s choice of treatment. In this open-label, randomized trial, 378 previously untreated HLA-A*02:01 positive patients with mUM were randomly allocate to either single-agent tebentafusp or investigator’s choice of single-agent pembrolizumab, ipilimumab, or dacarbazine. Dual checkpoint inhibition was not included in the control arm, because the trial was designed before publication of data from GEM-1402 and CA184-187. Compared to the control arm, tebentafusp produced a higher ORR (5% vs. 11%) and a significantly prolonged mOS (21.6 vs. 16.9 months, HR for death: 0.68, 95%CI: 0.540.87) and 3-year OS of (27% vs. 18%) [105]. Regarding safety, grade 3–4 TRAEs were more common in the experimental arm compared to the control group (44% vs. 17%). Although the OS benefit was statistically and clinically significant, the PFS benefit was marginal despite being statistically significant (3.4 vs. 2.9 months, HR for progression: 0.76; 95%CI: 0.60 to 0.97). Interestingly, the radiographic tumor response, as defined per RECIST criteria, was not directly associated with the survival benefit observed in patients. Patients with disease progression as the best response in the experimental arm had longer survival compared to patients with disease progression as the best response in the comparator arm. These findings suggest a clinically meaningful effect of tebentafusp in the absense of radiographically significant decrease in disease burden and highlight the need for surrogate prognostic factors. To this end, data from the single arm phase II trial of tebentafusp and updated data from the 3-year follow up of IMCgp100-202 indicate a statistically significant association between lower baseline ctDNA levels, early ctDNA clearance (mOS of 29.6 vs. 10.2 months) and > 50% reduction in ctDNA levels (HR for death: 0.41; 95%CI: 0.25–0.67) with overall survival [105].

Furthermore, current data exist regarding the effect of the sequence of administration of ICI and tebentafusp on the outcomes of patients suggest that ICI first results in better outcomes. In a retrospective study by Dimitriou et al. including 131 mUM patients, the ICI-first strategy resulted in significantly greater mOS (33.6 vs. 22.4 months, *P* = 0.004, respectively) and mPFS (20.3 vs. 12.0 months, P = 0.04, respectively) compared to tebentafusp-first [106]. Similarly, in the study of Tomsitz et al. in 78 patients with mUM, the mOS appeared to be longer, although not clincially significant, in ICI-first patients compared to the reversed treatment strategy (28.0 vs 24. Months) [107]. To the contrary, based on a small retrospective cohort of 20 patients, Koch et al. reported that the mPFS and mOS were longer in the group receiving tebentafusp first, compared to those receiving ICI first (2.8 vs. 2.3 months and 21.4 vs. 16.8 months) [108].

IMC-F106 C, another ImmTAC, which targets PRAME, an HLA-A*02:01-restricted epitope known to be present in UM [40], is currently being evaluated as a monotherapy or in combination with ICIs in a phase I trial for PRAME-positive solid tumors, including mUM (NCT04262466).

### Targeted treatment

Mitogen-activated protein kinase (MAPK) pathway activation is a frequent phenomenon in UM, primarily independent of *RAS* and *BRAF* upstream mutations [109, 110]. Preclinical evidence demonstrating the anti-melanoma effects of MAPK pathway inhibition at the level of MEK1/2 enzymes provided the rationale for the evaluation of MEK inhibitors in clinical trial settings [111, 112]. Selumetinib, a MEK1/2 inhibitor, conferred significantly improved clinical activity compared to dacarbazine or temozolomide in a randomized phase II trial with a total enrollment of 120 cases [113]. Selumetinib achieved an ORR of 14% versus 0%, a mOS of 11.8 versus 9.1 months and a mPFS of 15.9 versus 7 weeks, respectively (HR 0.46; 95% CI, 0.30–0.71). Furthermore, selumetinib was tested alone or in combination with paclitaxel in 77 chemotherapy naïve mUM patients in the phase II study SelPac (NCT. The combination was associated with higher ORR (14% vs. 4%), longer mPFS (4.8 vs. 3.4 months), but lower mOS (9 vs. 10 months) compared to single-agent selumetinib [114]. Acquisition of resistance to MEK inhibition arises due to adaptive GPCR-mediated YAP activation and AKT signaling activation, limiting the clinical utility of MEK inhibitors as monotherapy [115]. Cotreatment panobinostat, a histone deacetylase inhibitor, with the MEK inhibitor, trametinib, effectively suppressed the adaptive AKT and YAP signaling, leading to enhanced effects of MEK inhibition and significantly more profound uveal melanoma regression in vivo, compared to signle-agent trametinib [115]. To this end, binimetinib, a MEK inhibitor, is currently being evaluated in combination with belinostat, a histone deacetylase (HDAC) inhibitor, in a phase II study (NCT05170334) and with darovasertib in a phase I/II study (NCT03947385). Of note, however, negative results were observed in a phase II study (NCT03417739) with ulixertinib, an ERK1/2 inhibitor, as best response was stable disease in 4 out of 13 enrolled patients [116].

Another potential strategy is the inhibition of protein kinase C (PKC), which demonstrated promising preclinical anticancer effects, alone or in combination with MEK inhibition in GNAQ mutated UM [117]. In melanoma cell lines, Park et al. showed that MAPK, but not PI3 K/AKT signalling, was inhibited early upon PKC inhibition [118]. In a phase I trial (NCT02601378) with 68 mUM patients, Darovasertib, a PKC inhibitor, achieved an ORR of 9.1% (1 CR and 5 PR), while 45 patients experienced SD as their best response (DCR of 75%) [119]. The median duration of response was 10.1 months and the estimated 12-month PFS rate was 11.8%. With regards to safety, 42.6% of patients experienced grade 3/4 TRAEs. Furthermore, Darovasertib is also being evaluated in combination with crizotinib in pretreated and treatment naïve mUM patients, in a phase I/II basket study (NCT03947385). Interestingly, preliminary data showcase clinical efficacy that appears superior to current standards of care along with a manageable safety profile. At data cutoff, the PR rate was 30% in the pretreated and 45% in the treatment naïve group, while the mPFS was 7 months in both groups; antineoplastic effects were observed in both HLA-A2 positive and negative patients [120]. Based on these data, darovasertib plus crizotinib are currently being tested as first-line therapy versus investigator’s choice of treatment for HLA-A*02:01-negative mUM patients in a large, randomized phase II/III study (NCT05987332). Lastly, according to early data from the phase II NADOM trial (NCT02601378), darovasertib also produced promising effects in the neoadjuvant setting, inducing clinically meaningful UM shrinkage and enabling globe salvation and conversion to radiotherapy in six out of nine patients, who otherwise would require enucleation [121]. A larger phase II study (NCT05907954) is now in progress to further investigate the role of neoadjuvant/adjuvant darovasertib in patients with localized ocular melanoma.

The PI3 K/AKT pathway is another highly active signaling pathay in the majority of UMs.Ιn UM, PI3 Kδ signaling plays a role in tumor progression by promoting cell survvial, proliferation and apoptosis evasion. Furthermore, PI3 K/AKT signaling contributes to resistance to PKC inhibition in UM cell lines [118]. IOA-244 (roginolisib) blocks the activity of PI3 Kδ-dependent signalling, in both tumor cells and Tregs. Based on preliminary data from the phase I study (NCT04328844) in various tumors, including UM, roginolisib conferred a DCR of 75% and a mOS of 20.8 months [122]. Roginolisib is also being tested in comparison to investigator’s choice of care in patients with mUM in a phase II study (NCT06717126).

Mutations in *GNAQ* or *GNA11* drive oncogenesis in UM by inducing dephosphorylation and subsequent activation of Yes-associated protein 1 (YAP) and trasncription co-activator with PDZ-binding motif (TAZ), both of which are key effectors of the Hippo signalling pathway (Hippo-YAP-TAZ) [123]. Given its role in mediating YAP activation, focal adhesion kinase (FAK) has emerged as a promising therapeutic target. Preclinical studies have shown that FAK inhibition effectively supresses UM growth, both as monotherapy and in combination with MEK inhibition [124, 125]. In clinical trial settings, defactinib, a FAK inhibitor plus VS-6766, a RAF/MEK inhibitor, achieved a mPFS of 2.6 months and a mOS of 18.4 months in patients with mUM [126]. FAK inhibition (IN10018) alone or in combination with the MEK inhibitor cobimetinib is currently being investigated in a phase I study (NCT04109456). Interestingly, statin administration has been shown to counteract YAP nuclear localization, thereby inhibiting YAP activity [127]. To this end, cerivastatin plus trametinib, a MEK inhibitor, successfully reduced tumor cell proliferation and induced apoptosis in UM xenograft models [128]. Nonetheless, additional clinical trials are needed to confirm the efficacy of these strategies.

Hepatocyte growth factor (HGF)/mesenchymal-epithelial transition factor (MET) signaling is implicated in tumorigenesis and metastatic progression in several types of malignancies, including UM, where MET protein expression is detected in 86% of specimens [129]. In UM, the HGF/MET signaling pathway exerts pleiotropic protumor functions, including induction of epithelial-to-mesenchymal transition and promotion of tumor invasion and metastasis [129]. Crizotinib, a type I MET inhibitor, although ineffective as adjuvant therapy [130] is currently under investigation in combination with darovasertib in a phase I/II study (NCT03947385). Merestinib is a type II MET inhibitor with demonstrated activity against different kinases, including AXL, Tek and MKNK1/2. In mUM cells, merestinib effectively reversed HGF-mediated resistance to CDK4/6 inhibition, while CD4/6 inhibition plus HGF inhibition resulted in significantly greater tumor inhibition compared to either monotherapy in UM mouse models [131]. To this end, merestinib was evaluated alone or in combinatorial regimens in a phase I study in patients with advanced malignancies, including UM; in the UM cohort the reported 6-month PFS rate was 20% [132].

Lastly, c-KIT mutations are a relatively uncommon phenomenon in UM, in line with the generally poor outcomes of c-KIT-targeted agents [133]. To this imatinib failed to produce clinically meaningful results in mUM patients with KIT overexpression, yielding an ORR of 8%, a mOS of 7.5 months and a mPFS of 3.0 months [134]; no ORRs were reported in other clinical trials [135, 136]. Similar unimpressive findings have been observed with sunitinib. In a phase II study (NCT75033520), sunitib was tested as first-line versus dacarbazine and yielded no ORs compared to an ORR of 8% with dacarbazine and achieved a comparable mPFS and mOS to dacarbazine [65].

### Epigenetic strategies

Epigenetic alterations can result in aberrant gene regulation, thus playing an important role in tumorigenesis. Several strategies aimed at modulating these epigenetic mechanisms are currently under investigation, offering promising avenues for treatment.

Approximately half of primary UM cases display biallelic inactivation of *BAP1*, which is associated with more aggressive and metastatic disease [20]. In preclinical models of cultured UM cells, HDAC inhibition was found to switch the phenotypic effects of BAP1 loss, induce apoptosis and reverse UM cells’ profile to a differentiated, melanocytic gene expression pattern [137]. In the PEMDAC phase II clinical trial (NCT02697630) the HDAC inhibitor entinostat was combined with pembrolizumab in patients with mUM and demonstrated an ORR of 14%, a mPFS duration of 2.1 months and a mOS of 13.4 months, while maintaining a manageable toxicity profile [138]. Additionally, another phase II trial (NCT01587352) is assessing vorinostat, another HDAC inhibitor, in patients with mUM and results are awaited.

Beyond histone modifications, aberrant RNA splicing is emerging as a key epigenetic vulnerability in UM. *SF3B1* mutations, known to generate cryptic 3’ splice sites and neoantigens, may theoretically enhance tumor immunogenicity; however, clinical data have not yet demostrated an increased response to ICIs [139]. In leukemia models harboring mutations in splicing factors such as SF3B1, inhibition of protein arginine methyltransferases (PRMTs) has shown promising anti-tumor activity [140]. In UM, the PRMT5 inhibitor, PRT543, downregulates *SF3B1* target genes, which are involved in intron retention in *SF3B1* mutant cells. Combining PRT543 with DNA-alkylating agents or PARP inhibitors has demonstrated synergistic anti-tumor effects [141]. This therapeutic strategy is currently being explored in a phase I clinical trial (NCT03886831) for patients with UM.

Another epigenetic target under investigation is the bromodomain and extra-terminal (BET) protein family (BRD2, BRD3, BRD4, BRDT), which modulates chromatin structure and transcription by recognizing acetylated lysine residues on histones. In malignancies, BET proteins regulate the transcription of key oncogenes, making them attractive therapeutic targets [142]. Preclinical melanoma models have demonstrated that BRD4 knockout induces cell-cycle arrest and downregulates critical cell-cycle genes, underscoring the potential of BET inhibition [143]. PLX2853, a novel BRD4 inhibitor, is currently being assessed in a phase I/II trial (NCT03297424) involving patients with advanced tumors, including mUM.

PHP: percutaneous hepatic perfusion; SIRT: selective internal radiation therapy; LDT: liver-direct treatment; TIL: tumor infiltrating lymphocytes; ADC: antibody–drug conjugates; BAC: best alternative care; mAb: monoclonal antibody; TKI: tyrosine kinase inhibitor.

## Conclusions

UM remains a formidable clinical challenge due to its unique molecular characteristics, high metastatic potential, and limited response to conventional systemic therapies. While significant progress has been made in understanding the genetic and molecular drivers of UM, this knowledge has not been translated into a substantial survival benefit for patients with metastatic disease. Despite a range of effective local treatment options, nearly half of UM patients ultimately develop metastases, highlighting the urgent need for novel therapeutic strategies capable of altering the natural history of the disease. The recent approval of tebentafusp represents a landmark achievement in UM treatment, demonstrating a significant overall survival advantage in HLA-A*02:01-positive patients. Furthermore, promising experimental combinations have demonstrated significant clinical activity in late phase clinical trials, offering hope for further improvement in patient survival.

The emergence of genomic and transcriptomic profiling has provided invaluable insights into the molecular landscape of UM, refining prognostic classification and offering new avenues for targeted and personalized therapy. Future research should focus on leveraging these molecular insights to develop additional rational combination therapies, integrating immune-based strategies with targeted agents and epigenetic modulators to overcome tumor resistance. Ultimately, the path forward in UM management requires a multidisciplinary and collaborative approach, incorporating advances in molecular oncology and personalizing patient care.

## Key References

- Hassel, J.C.; Piperno-Neumann, S.; Rutkowski, P.; Baurain, J.F.; Schlaak, M.; Butler, M.O.; Sullivan, R.J.; Dummer, R.; Kirkwood, J.M.; Orloff, M.; et al. Three-Year Overall Survival with Tebentafusp in Metastatic Uveal Melanoma. *N Engl J Med* **2023**, *389*, 2256-2266,10.1056/NEJMoa2304753.
  - Updated, three-year outcomes of the IMCgp100-202 phase III trial based on which Tebentafusp received regulatory approval in metastatic Uveal Melanoma.
- Dimitriou, F.; Orloff, M.M.; Koch Hein, E.C.; Cheng, P.F.; Hughes, I.F.; Simeone, E.; Montazeri, K.; Grover, P.; Mehmi, I.; Gerard, C.L.; et al. Treatment sequence with tebentafusp and immune checkpoint inhibitors in patients with metastatic uveal melanoma and metastatic GNA11/GNAQ mutant melanocytic tumors. *Eur J Cancer* **2025**, *214*, 115161,10.1016/j.ejca.2024.115161.
  - This study reported retrospective data on the clinical outcome of tebentafusp followed by ICI or the inverse sequence. First-line tebentafusp was associated with significantly better outcomes.
- McKean, M.; Chmielowski, B.; Butler, M.O.; Carvajal, R.; Rodon, J.; Carlino, M.; Kim, K.B.; Wise-draper, T.; Khan, S.; Salama, A.K.S.; et al. 1081O ctDNA reduction and clinical efficacy of the darovasertib + crizotinib (daro + crizo) combination in metastatic uveal melanoma (MUM). *Annals of Oncology* **2023**, *34*, S651,10.1016/j.annonc.2023.09.2215.
  - This clinical study demonstrated promising clinical utility with darovasertib plus crizotinib both in pretreated and treatment naïve patients as well as HLA-A2 negative and positive cases. Based on these results, this combination is currently being investigated in a phase II/III study.
- Ziogas, D.C.; Theocharopoulos, C.; Koutouratsas, T.; Haanen, J.; Gogas, H. Mechanisms of resistance to immune checkpoint inhibitors in melanoma: What we have to overcome? *Cancer Treat Rev* **2022**, *113*, 102499, 10.1016/j.ctrv.2022.102499.
  - Single-agent CTLA-4 or PD-1 inhibition is associated with very limited clinical activity in metastatic uveal melanoma, owing to primary or acquired resistance mechanisms. This review provides a comprehensive analysis of the primary and secondary resistance phenomena.
- Olofsson Bagge, R.; Nelson, A.; Shafazand, A.; All-Eriksson, C.; Cahlin, C.; Elander, N.; Gustavsson, A.; Helgadottir, H.; Kiilgaard, J.F.; Kinhult, S.; et al. Survival and Quality of Life after Isolated Hepatic Perfusion with Melphalan as a Treatment for Uveal Melanoma Liver Metastases—Final Results from the Phase III Randomized Controlled Trial SCANDIUM. *Ann Surg* **2024**, 10.1097/SLA.0000000000006255.
  - The SCANDIUM trial compared isolated hepatic perfusion with melphalan to best alternative care and reported significantly better outcomes with IHP, providing further evidence on the efficacy of locoregional therapies in isolated hepatic metastases.
- Zager, J.S.; Orloff, M.; Ferrucci, P.F.; Choi, J.; Eschelman, D.J.; Glazer, E.S.; Ejaz, A.; Howard, J.H.; Richtig, E.; Ochsenreither, S.; et al. Efficacy and Safety of the Melphalan/Hepatic Delivery System in Patients with Unresectable Metastatic Uveal Melanoma: Results from an Open-Label, Single-Arm, Multicenter Phase 3 Study. *Ann Surg Oncol* **2024**, *31*, 5340–5351, https://doi.org/10.1245/s10434-024–15293-x.
  - Similarly to the SCANDIUM trial, FOCUS trial demonstrated superior efficacy of percutaneous hepatic perfusion with melphalan compared to systemic treatment.

## Data Availability

No datasets were generated or analysed during the current study.

## Abbreviations

CM: Cutaneous melanoma
UM: Uveal melanoma
mPFS: Median progression free survival
mOS: Median overall survival
ORR: Objective response rate
DCR: Disease control rate
ICI: Immune checkpoint inhibitor
HR: Hazard ratio
LUMPO: Liverpool uveal melanoma prognosticator online
TMB: Tumor mutational burden
MAA: Melanoma-associated antigens
FDA: Food and Drug Administration
EMA: European Medicines Agency
NCCN: National Comprehensive Cancer Network
COMS: Collaborative Ocular Melanoma Study
PD-1: Programmed cell death protein 1
GM-CSF: Granulocyte–macrophage colony-stimulating factor
CTLA-4: Cytotoxic T-lymphochyte-associated antigen 4
MAPK: Mitogen-activated protein kinase
LITT: Laser-induced interstitial thermotherapy
HDAC: Histone deacetylase
PHP: Percutaneous hepatic perfusion
TACE: Transarterial chemoembolization

## References

[1] Chang AE. Karnell LH. Menck HR. The National Cancer Data Base report on cutaneous and noncutaneous melanoma: a summary of 84,836 cases from the past decade. The American College of Surgeons Commission on Cancer and the American Cancer Society. Cancer 1998;83: 1664–1678. 10.1002/(sici)1097-0142(19981015)83:8<1664::aid-cncr23>3.0.co;2-g. 10.1002/(sici)1097-0142(19981015)83:8<1664::aid-cncr23>3.0.co;2-g 9781962

[2] Shields CL, Furuta M, Thangappan A, Nagori S, Mashayekhi A, Lally DR, Kelly CC, Rudich DS, Nagori AV, Wakade OA, et al. Metastasis of uveal melanoma millimeter-by-millimeter in 8033 consecutive eyes. Arch Ophthalmol. 2009;127:989–98. 10.1001/archophthalmol.2009.208. 19667335 10.1001/archophthalmol.2009.208

[3] Silva-Rodriguez P. Fernandez-Diaz D. Bande M. Pardo M. Loidi L. Blanco-Teijeiro MJ. GNAQ and GNA11 Genes: A Comprehensive Review on Oncogenesis, Prognosis and Therapeutic Opportunities in Uveal Melanoma. Cancers (Basel) 2022;14 10.3390/cancers14133066. 10.3390/cancers14133066 PMC9264989 35804836

[4] Kolandjian NA, Wei C, Patel SP, Richard JL, Dett T, Papadopoulos NE, Bedikian AY. Delayed systemic recurrence of uveal melanoma. Am J Clin Oncol. 2013;36:443–9. 10.1097/COC.0b013e3182546a6b. 22706174 10.1097/COC.0b013e3182546a6b PMC4574291

[5] Beasley AB, Chen FK, Isaacs TW, Gray ES. Future perspectives of uveal melanoma blood based biomarkers. Br J Cancer. 2022;126:1511–28. 10.1038/s41416-022-01723-8. 35190695 10.1038/s41416-022-01723-8 PMC9130512

[6] Khoja L, Atenafu EG, Suciu S, Leyvraz S, Sato T, Marshall E, Keilholz U, Zimmer L, Patel SP, Piperno-Neumann S, et al. Meta-analysis in metastatic uveal melanoma to determine progression free and overall survival benchmarks: an international rare cancers initiative (IRCI) ocular melanoma study. Ann Oncol. 2019;30:1370–80. 10.1093/annonc/mdz176. 31150059 10.1093/annonc/mdz176

[7] Collaborative Ocular Melanoma Study, G. Assessment of metastatic disease status at death in 435 patients with large choroidal melanoma in the Collaborative Ocular Melanoma Study (COMS): COMS report no. 15. Arch Ophthalmol 2001;119:670–676, 10.1001/archopht.119.5.670. 10.1001/archopht.119.5.670 11346394

[8] Klempner SJ, Fabrizio D, Bane S, Reinhart M, Peoples T, Ali SM, Sokol ES, Frampton G, Schrock AB, Anhorn R, et al. Tumor Mutational Burden as a Predictive Biomarker for Response to Immune Checkpoint Inhibitors: A Review of Current Evidence. Oncologist. 2020;25:e147–59. 10.1634/theoncologist.2019-0244. 31578273 10.1634/theoncologist.2019-0244 PMC6964127

[9] Hu Z, Ott PA, Wu CJ. Towards personalized, tumour-specific, therapeutic vaccines for cancer. Nat Rev Immunol. 2018;18:168–82. 10.1038/nri.2017.131. 29226910 10.1038/nri.2017.131 PMC6508552

[10] McGrail DJ, Pilie PG, Rashid NU, Voorwerk L, Slagter M, Kok M, Jonasch E, Khasraw M, Heimberger AB, Lim B, et al. High tumor mutation burden fails to predict immune checkpoint blockade response across all cancer types. Ann Oncol. 2021;32:661–72. 10.1016/j.annonc.2021.02.006. 33736924 10.1016/j.annonc.2021.02.006 PMC8053682

[11] Chalmers ZR, Connelly CF, Fabrizio D, Gay L, Ali SM, Ennis R, Schrock A, Campbell B, Shlien A, Chmielecki J, et al. Analysis of 100,000 human cancer genomes reveals the landscape of tumor mutational burden. Genome Med. 2017;9:34. 10.1186/s13073-017-0424-2. 28420421 10.1186/s13073-017-0424-2 PMC5395719

[12] Pfeifer GP. Mechanisms of UV-induced mutations and skin cancer. Genome Instab Dis. 2020;1:99–113. 10.1007/s42764-020-00009-8. 34589668 10.1007/s42764-020-00009-8 PMC8477449

[13] Dousset L. Poizeau F. Robert C. Mansard S. Mortier L. Caumont C. Routier É. Dupuy A. Rouanet J. Battistella M. et al. Positive Association Between Location of Melanoma, Ultraviolet Signature, Tumor Mutational Burden, and Response to Anti–PD-1 Therapy. JCO Prec Onco 2021;1821–1829, 10.1200/po.21.00084. 10.1200/PO.21.00084 PMC8691497 34950838

[14] Hilke FJ. Sinnberg T. Gschwind A. Niessner H. Demidov G. Amaral T. Ossowski S. Bonzheim, I. Rocken M. Riess O et al. Distinct Mutation Patterns Reveal Melanoma Subtypes and Influence Immunotherapy Response in Advanced Melanoma Patients. Cancers (Basel) 2020;12 10.3390/cancers12092359. 10.3390/cancers12092359 PMC7563780 32825510

[15] Mobuchon L. Battistella A. Bardel C. Scelo G. Renoud A. Houy A. Cassoux N. Milder M. Cancel-Tassin G. Cussenot O. et al. A GWAS in uveal melanoma identifies risk polymorphisms in the CLPTM1L locus. 10.1038/s41525-017-0008-5 PMC5542017 28781888

[16] Abdel-Rahman MH, Pilarski R, Cebulla CM, Massengill JB, Christopher BN, Boru G, Hovland P, Davidorf FH. Germline BAP1 mutation predisposes to uveal melanoma, lung adenocarcinoma, meningioma, and other cancers. J Med Genet. 2011;48:856–9. 10.1136/jmedgenet-2011-100156. 21941004 10.1136/jmedgenet-2011-100156 PMC3825099

[17] Rai K, Pilarski R, Boru G, Rehman M, Saqr AH, Massengill JB, Singh A, Marino MJ, Davidorf FH, Cebulla CM, et al. Germline BAP1 alterations in familial uveal melanoma. Genes Chromosomes Cancer. 2017;56:168–74. 10.1002/gcc.22424. 27718540 10.1002/gcc.22424 PMC5490375

[18] Li Y, Shi J, Yang J, Ge S, Zhang J, Jia R, Fan X. Uveal melanoma: progress in molecular biology and therapeutics. Ther Adv Med Oncol. 2020;12:1758835920965852. 10.1177/1758835920965852. 33149769 10.1177/1758835920965852 PMC7586035

[19] Kobrinski DA, Yang H, Kittaneh M. BAP1: role in carcinogenesis and clinical implications. Transl Lung Cancer Res. 2020;9:S60–6. 10.21037/tlcr.2019.11.24. 32206571 10.21037/tlcr.2019.11.24 PMC7082261

[20] Koopmans AE, Verdijk RM, Brouwer RW, van den Bosch TP, van den Berg MM, Vaarwater J, Kockx CE, Paridaens D, Naus NC, Nellist M, et al. Clinical significance of immunohistochemistry for detection of BAP1 mutations in uveal melanoma. Mod Pathol. 2014;27:1321–30. 10.1038/modpathol.2014.43. 24633195 10.1038/modpathol.2014.43

[21] Martin M, Masshofer L, Temming P, Rahmann S, Metz C, Bornfeld N, van de Nes J, Klein-Hitpass L, Hinnebusch AG, Horsthemke B, et al. Exome sequencing identifies recurrent somatic mutations in EIF1AX and SF3B1 in uveal melanoma with disomy 3. Nat Genet. 2013;45:933–6. 10.1038/ng.2674. 23793026 10.1038/ng.2674 PMC4307600

[22] Furney SJ, Pedersen M, Gentien D, Dumont AG, Rapinat A, Desjardins L, Turajlic S, Piperno-Neumann S, de la Grange P, Roman-Roman S, et al. SF3B1 mutations are associated with alternative splicing in uveal melanoma. Cancer Discov. 2013;3:1122–9. 10.1158/2159-8290.CD-13-0330. 23861464 10.1158/2159-8290.CD-13-0330 PMC5321577

[23] Yavuzyigitoglu, S.; Koopmans, A.E.; Verdijk, R.M.; Vaarwater, J.; Eussen, B.; van Bodegom, A.; Paridaens, D.; Kilic, E.; de Klein, A.; Rotterdam Ocular Melanoma Study, G. Uveal Melanomas with SF3B1 Mutations: A Distinct Subclass Associated with Late-Onset Metastases. *Ophthalmology* **2016**, *123*, 1118–1128, 10.1016/j.ophtha.2016.01.023. 10.1016/j.ophtha.2016.01.023 26923342

[24] Mobuchon L, Derrien AC, Houy A, Verrier T, Pierron G, Cassoux N, Milder M, Deleuze JF, Boland A, Scelo G, et al. Different Pigmentation Risk Loci for High-Risk Monosomy 3 and Low-Risk Disomy 3 Uveal Melanomas. J Natl Cancer Inst. 2022;114:302–9. 10.1093/jnci/djab167. 34424336 10.1093/jnci/djab167 PMC8826635

[25] Ewens KG, Kanetsky PA, Richards-Yutz J, Al-Dahmash S, De Luca MC, Bianciotto CG, Shields CL, Ganguly A. Genomic profile of 320 uveal melanoma cases: chromosome 8p-loss and metastatic outcome. Invest Ophthalmol Vis Sci. 2013;54:5721–9. 10.1167/iovs.13-12195. 23821189 10.1167/iovs.13-12195

[26] Bornfeld N, Prescher G, Becher R, Hirche H, Jöckel KH, Horsthemke B. Prognostic implications of monosomy 3 in uveal melanoma. The Lancet. 1996;347:1222–5. 10.1016/S0140-6736(96)90736-9. 10.1016/s0140-6736(96)90736-9 8622452

[27] Kaliki S, Shields CL, Shields JA. Uveal melanoma: estimating prognosis. Indian J Ophthalmol. 2015;63:93–102. 10.4103/0301-4738.154367. 25827538 10.4103/0301-4738.154367 PMC4399142

[28] Kaliki S, Shields CL. Uveal melanoma: relatively rare but deadly cancer. Eye (Lond). 2017;31:241–57. 10.1038/eye.2016.275. 27911450 10.1038/eye.2016.275 PMC5306463

[29] Onken MD, Worley LA, Ehlers JP, Harbour JW. Gene expression profiling in uveal melanoma reveals two molecular classes and predicts metastatic death. Cancer Res. 2004;64:7205–9. 10.1158/0008-5472.CAN-04-1750. 15492234 10.1158/0008-5472.CAN-04-1750 PMC5407684

[30] Onken MD, Worley LA, Char DH, Augsburger JJ, Correa ZM, Nudleman E, Aaberg TM Jr, Altaweel MM, Bardenstein DS, Finger PT, et al. Collaborative Ocular Oncology Group report number 1: prospective validation of a multi-gene prognostic assay in uveal melanoma. Ophthalmology. 2012;119:1596–603. 10.1016/j.ophtha.2012.02.017. 22521086 10.1016/j.ophtha.2012.02.017 PMC3404209

[31] McLean MJ, Foster WD, Zimmerman LE. Prognostic factors in small malignant melanomas of choroid and ciliary body. Arch Ophthalmol. 1977;95:48–58. 10.1001/archopht.1977.04450010050004. 836203 10.1001/archopht.1977.04450010050004

[32] de Bruyn DP, van Poppelen NM, Brands T, van den Boom SC, Eikenboom E, Wagner A, van Veghel-Plandsoen MM, Geeven G, Beverloo B, van Rij CM, et al. Evaluation of Circulating Tumor DNA as a Liquid Biomarker in Uveal Melanoma. Invest Ophthalmol Vis Sci. 2024;65:11. 10.1167/iovs.65.2.11. 38319670 10.1167/iovs.65.2.11 PMC10854420

[33] Carvajal RD, Butler MO, Shoushtari AN, Hassel JC, Ikeguchi A, Hernandez-Aya L, Nathan P, Hamid O, Piulats JM, Rioth M, et al. Clinical and molecular response to tebentafusp in previously treated patients with metastatic uveal melanoma: a phase 2 trial. Nat Med. 2022;28:2364–73. 10.1038/s41591-022-02015-7. 36229663 10.1038/s41591-022-02015-7 PMC9671803

[34] Rodrigues M, Ramtohul T, Rampanou A, Sandoval JL, Houy A, Servois V, Mailly-Giacchetti L, Pierron G, Vincent-Salomon A, Cassoux N, et al. Prospective assessment of circulating tumor DNA in patients with metastatic uveal melanoma treated with tebentafusp. Nat Commun. 2024;15:8851. 10.1038/s41467-024-53145-0. 39402032 10.1038/s41467-024-53145-0 PMC11473804

[35] Dogrusöz M, Bagger M, van Duinen SG, Kroes WG, Ruivenkamp CA, Böhringer S, Andersen KK, Luyten GP, Kiilgaard JF, Jager MJ. The Prognostic Value of AJCC Staging in Uveal Melanoma Is Enhanced by Adding Chromosome 3 and 8q Status. Invest Ophthalmol Vis Sci. 2017;58:833–42. 10.1167/iovs.16-20212. 28159971 10.1167/iovs.16-20212

[36] Shields CL, Dockery PW, Mayro EL, Bas Z, Yaghy A, Lally SE, Orloff M, Sato T, Shields JA. Conditional survival of uveal melanoma using The Cancer Genome Atlas (TCGA) classification (Simplified Version) in 1001 cases. Saudi J Ophthalmol. 2022;36:308–14. 10.4103/sjopt.sjopt_69_21. 36276251 10.4103/sjopt.sjopt_69_21 PMC9583357

[37] DeParis SW, Taktak A, Eleuteri A, Enanoria W, Heimann H, Coupland SE, Damato B. External Validation of the Liverpool Uveal Melanoma Prognosticator Online. Invest Ophthalmol Vis Sci. 2016;57:6116–22. 10.1167/iovs.16-19654. 27835710 10.1167/iovs.16-19654

[38] Vaquero-Garcia J, Lalonde E, Ewens KG, Ebrahimzadeh J, Richard-Yutz J, Shields CL, Barrera A, Green CJ, Barash Y, Ganguly A. PRiMeUM: A Model for Predicting Risk of Metastasis in Uveal Melanoma. Invest Ophthalmol Vis Sci. 2017;58:4096–105. 10.1167/iovs.17-22255. 28828481 10.1167/iovs.17-22255 PMC6108308

[39] Carvajal RD, Sacco JJ, Jager MJ, Eschelman DJ, Olofsson Bagge R, Harbour JW, Chieng ND, Patel SP, Joshua AM, Piperno-Neumann S. Advances in the clinical management of uveal melanoma. Nat Rev Clin Oncol. 2023;20:99–115. 10.1038/s41571-022-00714-1. 36600005 10.1038/s41571-022-00714-1

[40] Field MG, Decatur CL, Kurtenbach S, Gezgin G, van der Velden PA, Jager MJ, Kozak KN, Harbour JW. PRAME as an Independent Biomarker for Metastasis in Uveal Melanoma. Clin Cancer Res. 2016;22:1234–42. 10.1158/1078-0432.ccr-15-2071. 26933176 10.1158/1078-0432.CCR-15-2071 PMC4780366

[41] Harbour JW. Correa ZM. Schefler AC. Mruthyunjaya P. Materin MA. Aaberg TA. Skalet AH. Reichstein DA. Weis E. Kim IK et al. 15-Gene Expression Profile and PRAME as Integrated Prognostic Test for Uveal Melanoma: First Report of Collaborative Ocular Oncology Group Study No. 2 (COOG2.1). J Clin Oncol 2024;42:3319–3329 10.1200/JCO.24.00447. 10.1200/JCO.24.00447 PMC11421563 39052972

[42] Field MG, Durante MA, Decatur CL, Tarlan B, Oelschlager KM, Stone JF, Kuznetsov J, Bowcock AM, Kurtenbach S, Harbour JW. Epigenetic reprogramming and aberrant expression of PRAME are associated with increased metastatic risk in Class 1 and Class 2 uveal melanomas. Oncotarget. 2016;7:59209–19. 10.18632/oncotarget.10962. 27486988 10.18632/oncotarget.10962 PMC5312306

[43] Mariani P, Piperno-Neumann S, Servois V, Berry MG, Dorval T, Plancher C, Couturier J, Levy-Gabriel C, Lumbroso-Le Rouic L, Desjardins L, et al. Surgical management of liver metastases from uveal melanoma: 16 years’ experience at the Institut Curie. Eur J Surg Oncol. 2009;35:1192–7. 10.1016/j.ejso.2009.02.016. 19329272 10.1016/j.ejso.2009.02.016

[44] Trivedi DB, Aldulaimi N, Karydis I, Wheater M, Modi S, Stedman B, Karavias D, Primrose J, Pearce N, Takhar AS. Liver resection for metastatic uveal melanoma: experience from a supra-regional centre and review of literature. Melanoma Res. 2023;33:71–9. 10.1097/CMR.0000000000000867. 36409208 10.1097/CMR.0000000000000867

[45] Rivoire M, Kodjikian L, Baldo S, Kaemmerlen P, Negrier S, Grange JD. Treatment of liver metastases from uveal melanoma. Ann Surg Oncol. 2005;12:422–8. 10.1245/ASO.2005.06.032. 15886904 10.1245/ASO.2005.06.032

[46] Salmon RJ, Levy C, Plancher C, Dorval T, Desjardins L, Leyvraz S, Pouillart P, Schlienger P, Servois V, Asselain B. Treatment of liver metastases from uveal melanoma by combined surgery-chemotherapy. Eur J Surg Oncol. 1998;24:127–30. 10.1016/s0748-7983(98)91485-8. 9591028 10.1016/s0748-7983(98)91485-8

[47] Dueland S, Solheim J, Foss A, Grut H, Hagness M, Line P-D. Dismal survival following liver transplantation for liver-only metastases in patients with ocular malignant melanoma. Trends in Transplantation. 2020;2020:13. 10.15761/TiT.1000282.

[48] de Brito RV. Mancini MW. Palumbo MDN. Moraes LHO. Rodrigues GJ. Cervantes O. Sercarz JA. Paiva MB. The Rationale for "Laser-Induced Thermal Therapy (LITT) and Intratumoral Cisplatin" Approach for Cancer Treatment. Int J Mol Sci 2022;23 10.3390/ijms23115934. 10.3390/ijms23115934 PMC9180481 35682611

[49] Bale R, Schullian P, Schmuth M, Widmann G, Jaschke W, Weinlich G. Stereotactic Radiofrequency Ablation for Metastatic Melanoma to the Liver. Cardiovasc Intervent Radiol. 2016;39:1128–35. 10.1007/s00270-016-1336-z. 27055850 10.1007/s00270-016-1336-z

[50] Mariani P, Almubarak MM, Kollen M, Wagner M, Plancher C, Audollent R, Piperno-Neumann S, Cassoux N, Servois V. Radiofrequency ablation and surgical resection of liver metastases from uveal melanoma. Eur J Surg Oncol. 2016;42:706–12. 10.1016/j.ejso.2016.02.019. 26968227 10.1016/j.ejso.2016.02.019

[51] Eichler K, Zangos S, Gruber-Rouh T, Vogl TJ, Mack MG. MR-guided laser-induced thermotherapy (LITT) in patients with liver metastases of uveal melanoma. J Eur Acad Dermatol Venereol. 2014;28:1756–60. 10.1111/jdv.12405. 24593299 10.1111/jdv.12405

[52] Connell LC, Kemeny NE. Intraarterial Chemotherapy for Liver Metastases. Surg Oncol Clin N Am. 2021;30:143–58. 10.1016/j.soc.2020.08.005. 33220802 10.1016/j.soc.2020.08.005 PMC8594481

[53] Sato T. Locoregional management of hepatic metastasis from primary uveal melanoma. Semin Oncol. 2010;37:127–38. 10.1053/j.seminoncol.2010.03.014. 20494705 10.1053/j.seminoncol.2010.03.014

[54] Melichar B, Voboril Z, Lojik M, Krajina A. Liver metastases from uveal melanoma: clinical experience of hepatic arterial infusion of cisplatin, vinblastine and dacarbazine. Hepatogastroenterology. 2009;56:1157–62. 19760961

[55] Leyvraz S, Piperno-Neumann S, Suciu S, Baurain JF, Zdzienicki M, Testori A, Marshall E, Scheulen M, Jouary T, Negrier S, et al. Hepatic intra-arterial versus intravenous fotemustine in patients with liver metastases from uveal melanoma (EORTC 18021): a multicentric randomized trial. Ann Oncol. 2014;25:742–6. 10.1093/annonc/mdt585. 24510314 10.1093/annonc/mdt585 PMC4433517

[56] Olofsson Bagge R, Nelson A, Shafazand A, All-Eriksson C, Cahlin C, Elander N, Gustavsson A, Helgadottir H, Kiilgaard JF, Kinhult S, et al. Survival and Quality of Life after Isolated Hepatic Perfusion with Melphalan as a Treatment for Uveal Melanoma Liver Metastases - Final Results from the Phase III Randomized Controlled Trial SCANDIUM. Ann Surg. 2024. 10.1097/SLA.0000000000006255. 38420778 10.1097/SLA.0000000000006255 PMC12140551

[57] Zager JS, Orloff M, Ferrucci PF, Choi J, Eschelman DJ, Glazer ES, Ejaz A, Howard JH, Richtig E, Ochsenreither S, et al. Efficacy and Safety of the Melphalan/Hepatic Delivery System in Patients with Unresectable Metastatic Uveal Melanoma: Results from an Open-Label, Single-Arm, Multicenter Phase 3 Study. Ann Surg Oncol. 2024;31:5340–51. 10.1245/s10434-024-15293-x. 38704501 10.1245/s10434-024-15293-x PMC11249544

[58] Carrasco CH. Wallace S. Charnsangavej C. Papadopoulos NE. Patt YZ. Mavligit GM. Treatment of hepatic metastases in ocular melanoma. Embolization of the hepatic artery with polyvinyl sponge and cisplatin. JAMA 1986;255:3152–3154. 3702027

[59] DePietro DM, Li X, Shamimi-Noori SM. Chemoembolization Beyond Hepatocellular Carcinoma: What Tumors Can We Treat and When? Semin Intervent Radiol. 2024;41:27–47. 10.1055/s-0043-1777716. 38495263 10.1055/s-0043-1777716 PMC10940046

[60] Dayani PN, Gould JE, Brown DB, Sharma KV, Linette GP, Harbour JW. Hepatic metastasis from uveal melanoma: angiographic pattern predictive of survival after hepatic arterial chemoembolization. Arch Ophthalmol. 2009;127:628–32. 10.1001/archophthalmol.2009.45. 19433711 10.1001/archophthalmol.2009.45

[61] Valpione S, Aliberti C, Parrozzani R, Bazzi M, Pigozzo J, Midena E, Pilati P, Campana LG, Chiarion-Sileni V. A retrospective analysis of 141 patients with liver metastases from uveal melanoma: a two-cohort study comparing transarterial chemoembolization with CPT-11 charged microbeads and historical treatments. Melanoma Res. 2015;25:164–8. 10.1097/CMR.0000000000000129. 25521594 10.1097/CMR.0000000000000129

[62] Yamamoto A, Chervoneva I, Sullivan KL, Eschelman DJ, Gonsalves CF, Mastrangelo MJ, Berd D, Shields JA, Shields CL, Terai M, et al. High-dose immunoembolization: survival benefit in patients with hepatic metastases from uveal melanoma. Radiology. 2009;252:290–8. 10.1148/radiol.2521081252. 19561263 10.1148/radiol.2521081252 PMC6944074

[63] Valsecchi ME, Terai M, Eschelman DJ, Gonsalves CF, Chervoneva I, Shields JA, Shields CL, Yamamoto A, Sullivan KL, Laudadio M, et al. Double-blinded, randomized phase II study using embolization with or without granulocyte-macrophage colony-stimulating factor in uveal melanoma with hepatic metastases. J Vasc Interv Radiol. 2015;26:523-532.e522. 10.1016/j.jvir.2014.11.037. 25678394 10.1016/j.jvir.2014.11.037 PMC4417549

[64] Peuker C-AA, Bucourt MD, Gebauer B, Amthauer H, Erxleben C, Eucker J, Keller U, Leyvraz S, Joussen AM, Keilholz U, et al. First interim analysis of the SirTac trial: A randomized phase II study of SIRT and DSM-TACE in patients with liver metastases from uveal melanoma. J Clin Oncol. 2022;40:9511–9511. 10.1200/JCO.2022.40.16_suppl.9511.

[65] Sacco JJ, Nathan PD, Danson S, Lorigan P, Nicholson S, Ottensmeier C, Corrie P, Steven N, Goodman A, Larkin JMG, et al. Sunitinib versus dacarbazine as first-line treatment in patients with metastatic uveal melanoma. J Clin Oncol. 2013;31:9031–9031. 10.1200/jco.2013.31.15_suppl.9031.

[66] Luke JJ, Olson DJ, Allred JB, Strand CA, Bao R, Zha Y, Carll T, Labadie BW, Bastos BR, Butler MO, et al. Randomized Phase II Trial and Tumor Mutational Spectrum Analysis from Cabozantinib versus Chemotherapy in Metastatic Uveal Melanoma (Alliance A091201). Clin Cancer Res. 2020;26:804–11. 10.1158/1078-0432.CCR-19-1223. 31558480 10.1158/1078-0432.CCR-19-1223 PMC7055933

[67] Branisteanu DC, Bogdanici CM, Branisteanu DE, Maranduca MA, Zemba M, Balta F, Branisteanu CI, Moraru AD. Uveal melanoma diagnosis and current treatment options (Review). Exp Ther Med. 2021;22:1428. 10.3892/etm.2021.10863. 34707709 10.3892/etm.2021.10863 PMC8543295

[68] Singh M, Durairaj P, Yeung J. Uveal Melanoma: A Review of the Literature. Oncol Ther. 2018;6:87–104. 10.1007/s40487-018-0056-8. 32700136 10.1007/s40487-018-0056-8 PMC7359963

[69] Schinzari G, Rossi E, Cassano A, Dadduzio V, Quirino M, Pagliara M, Blasi MA, Barone C. Cisplatin, dacarbazine and vinblastine as first line chemotherapy for liver metastatic uveal melanoma in the era of immunotherapy: a single institution phase II study. Melanoma Res. 2017;27:591–5. 10.1097/CMR.0000000000000401. 29076951 10.1097/CMR.0000000000000401

[70] Theocharopoulos C. Lialios PP. Samarkos M. Gogas H. Ziogas DC. Antibody-Drug Conjugates: Functional Principles and Applications in Oncology and Beyond. Vaccines (Basel) 2021;9: 10.3390/vaccines9101111. 10.3390/vaccines9101111 PMC8538104 34696218

[71] Theocharopoulos C, Ziogas IA, Douligeris C-C, Efstathiou A, Kolorizos E, Ziogas DC, Kontis E. Antibody-drug conjugates for hepato-pancreato-biliary malignancies: “Magic bullets” to the rescue? Cancer Treat Rev. 2024;129. 10.1016/j.ctrv.2024.102806. 39094332 10.1016/j.ctrv.2024.102806

[72] Theocharopoulos C, Lialios PP, Gogas H, Ziogas DC. An overview of antibody-drug conjugates in oncological practice. Ther Adv Med Oncol. 2020;12:1758835920962997. 10.1177/1758835920962997. 33088347 10.1177/1758835920962997 PMC7543133

[73] Sandhu S, McNeil CM, LoRusso P, Patel MR, Kabbarah O, Li C, Sanabria S, Flanagan WM, Yeh RF, Brunstein F, et al. Phase I study of the anti-endothelin B receptor antibody-drug conjugate DEDN6526A in patients with metastatic or unresectable cutaneous, mucosal, or uveal melanoma. Invest New Drugs. 2020;38:844–54. 10.1007/s10637-019-00832-1. 31385109 10.1007/s10637-019-00832-1

[74] Hasanov M. Rioth MJ. Kendra K. Hernandez-Aya L. Joseph RW. Williamson S. Chandra S. Shirai K. Turner CD. Lewis K et al. A Phase II Study of Glembatumumab Vedotin for Metastatic Uveal Melanoma. Cancers (Basel) 2020;12: 10.3390/cancers12082270. 10.3390/cancers12082270 PMC7465139 32823698

[75] Wk J. D'Alessio JA. Yerramilli-Rao P. Blankenship J. Buntin K. Burger M. Chen Z. Cho YS. Davis J. Ebel N et al. Abstract IA022: DYP688, a first-in-class PMEL17-targeted antibody drug conjugate delivering a Gq/11-specific inhibitor for the treatment of metastatic uveal melanoma. Mol Cancer Ther 2024;23:IA022-IA022, 10.1158/1538-8514.Cancerchem24-ia022.

[76] Ziogas DC, Theocharopoulos C, Koutouratsas T, Haanen J, Gogas H. Mechanisms of resistance to immune checkpoint inhibitors in melanoma: What we have to overcome? Cancer Treat Rev. 2022;113. 10.1016/j.ctrv.2022.102499. 36542945 10.1016/j.ctrv.2022.102499

[77] Danielli R, Ridolfi R, Chiarion-Sileni V, Queirolo P, Testori A, Plummer R, Boitano M, Calabrò L, Rossi CD, Giacomo AM, et al. Ipilimumab in pretreated patients with metastatic uveal melanoma: safety and clinical efficacy. Cancer Immunol Immunother. 2012;61:41–8. 10.1007/s00262-011-1089-0. 21833591 10.1007/s00262-011-1089-0 PMC11028946

[78] Zimmer L, Vaubel J, Mohr P, Hauschild A, Utikal J, Simon J, Garbe C, Herbst R, Enk A, Kämpgen E, et al. Phase II DeCOG-study of ipilimumab in pretreated and treatment-naïve patients with metastatic uveal melanoma. PLoS ONE. 2015;10. 10.1371/journal.pone.0118564. 25761109 10.1371/journal.pone.0118564 PMC4356548

[79] Rozeman EA, Prevoo W, Meier MAJ, Sikorska K, Van TM, van de Wiel BA, van der Wal JE, Mallo HA, Grijpink-Ongering LG, Broeks A, et al. Phase Ib/II trial testing combined radiofrequency ablation and ipilimumab in uveal melanoma (SECIRA-UM). Melanoma Res. 2020;30:252–60. 10.1097/cmr.0000000000000653. 31895753 10.1097/CMR.0000000000000653

[80] Karydis I, Chan PY, Wheater M, Arriola E, Szlosarek PW, Ottensmeier CH. Clinical activity and safety of Pembrolizumab in Ipilimumab pre-treated patients with uveal melanoma. Oncoimmunology. 2016;5. 10.1080/2162402x.2016.1143997. 27467964 10.1080/2162402X.2016.1143997 PMC4910726

[81] Rossi E, Pagliara MM, Orteschi D, Dosa T, Sammarco MG, Caputo CG, Petrone G, Rindi G, Zollino M, Blasi MA, et al. Pembrolizumab as first-line treatment for metastatic uveal melanoma. Cancer Immunol Immunother. 2019;68:1179–85. 10.1007/s00262-019-02352-6. 31175402 10.1007/s00262-019-02352-6 PMC6584707

[82] Joshua AM, Monzon JG, Mihalcioiu C, Hogg D, Smylie M, Cheng T. A phase 2 study of tremelimumab in patients with advanced uveal melanoma. Melanoma Res. 2015;25:342–7. 10.1097/CMR.0000000000000175. 26050146 10.1097/CMR.0000000000000175

[83] Piulats JM, Espinosa E, de la Cruz Merino L, Varela M, Alonso Carrión L, Martín-Algarra S, López Castro R, Curiel T, Rodríguez-Abreu D, Redrado M, et al. Nivolumab Plus Ipilimumab for Treatment-Naïve Metastatic Uveal Melanoma: An Open-Label, Multicenter, Phase II Trial by the Spanish Multidisciplinary Melanoma Group (GEM-1402). J Clin Oncol. 2021;39:586–98. 10.1200/jco.20.00550. 33417511 10.1200/JCO.20.00550

[84] Pelster MS, Gruschkus SK, Bassett R, Gombos DS, Shephard M, Posada L, Glover MS, Simien R, Diab A, Hwu P, et al. Nivolumab and Ipilimumab in Metastatic Uveal Melanoma: Results From a Single-Arm Phase II Study. J Clin Oncol. 2021;39:599–607. 10.1200/JCO.20.00605. 33125309 10.1200/JCO.20.00605 PMC8257877

[85] Najjar, Y.G.; Navrazhina, K.; Ding, F.; Bhatia, R.; Tsai, K.; Abbate, K.; Durden, B.; Eroglu, Z.; Bhatia, S.; Park, S.; et al. Ipilimumab plus nivolumab for patients with metastatic uveal melanoma: a multicenter, retrospective study. *J Immunother Cancer* **2020**, *8*, 10.1136/jitc-2019-000331. 10.1136/jitc-2019-000331 PMC7319717 32581057

[86] Heppt MV, Amaral T, Kahler KC, Heinzerling L, Hassel JC, Meissner M, Kreuzberg N, Loquai C, Reinhardt L, Utikal J, et al. Combined immune checkpoint blockade for metastatic uveal melanoma: a retrospective, multi-center study. J Immunother Cancer. 2019;7:299. 10.1186/s40425-019-0800-0. 31722735 10.1186/s40425-019-0800-0 PMC6854774

[87] Tong TML, Burgmans MC, Speetjens FM, van Erkel AR, van der Meer RW, van Rijswijk CSP, Jonker-Bos MA, Roozen CFM, Sporrel-Blokland M, Lutjeboer J, et al. Combining Melphalan Percutaneous Hepatic Perfusion with Ipilimumab Plus Nivolumab in Advanced Uveal Melanoma: First Safety and Efficacy Data from the Phase Ib Part of the Chopin Trial. Cardiovasc Intervent Radiol. 2023;46:350–9. 10.1007/s00270-022-03338-1. 36624292 10.1007/s00270-022-03338-1

[88] Patel SP, Haymaker C, Sheth RA, Kuban JD, Weintraub J, Wehrenberg-Klee E, Novelli P, Gonsalves C, Adamo R, Honaker V, et al. Abstract 5881: PERIO-01: Initial safety experience and immunologic effects of a Class C TLR9 agonist using pressure- enabled drug delivery in a phase 1 trial of hepatic arterial infusion of SD-101 +/- checkpoint inhibition in metastatic uveal melanoma. Can Res. 2023;83:5881–5881. 10.1158/1538-7445.Am2023-5881.

[89] Lutzky J, Hernandez-Aya L, Feun L, Correa Z, King J, Estevez C, Decatur C, Dollar JJ, Reis I, Harbour JW. 1126P A phase II study of nivolumab/relatlimab in metastatic uveal melanoma. Ann Oncol. 2024;35:S741. 10.1016/j.annonc.2024.08.1194.

[90] Natale, C.A.; Li, J.; Zhang, J.; Dahal, A.; Dentchev, T.; Stanger, B.Z.; Ridky, T.W. Activation of G protein-coupled estrogen receptor signaling inhibits melanoma and improves response to immune checkpoint blockade. *Elife* **2018**, *7*, 10.7554/eLife.31770. 10.7554/eLife.31770 PMC5770157 29336307

[91] Shoushtari A, Chaney M, Cohen J, Garyantes T, Lin J, Ishizuka J, Mita A, Mita M, Muller C, Natale C, et al. The effect of LNS8801 alone and in combination with pembrolizumab in patients with metastatic uveal melanoma. J Clin Oncol. 2023;41:9543–9543. 10.1200/JCO.2023.41.16_suppl.9543.

[92] Wu, L.; Maki, C.G. MDM2: RING Finger Protein and Regulator of p53. In *Zinc Finger Proteins: From Atomic Contact to Cellular Function*, Iuchi, S., Kuldell, N., Eds.; Springer US: Boston, MA, 2005; pp. 252–260.

[93] Fang DD, Tang Q, Kong Y, Wang Q, Gu J, Fang X, Zou P, Rong T, Wang J, Yang D, et al. MDM2 inhibitor APG-115 synergizes with PD-1 blockade through enhancing antitumor immunity in the tumor microenvironment. J Immunother Cancer. 2019;7:327. 10.1186/s40425-019-0750-6. 31779710 10.1186/s40425-019-0750-6 PMC6883539

[94] McKean M, Tolcher AW, Reeves JA, Chmielowski B, Shaheen MF, Beck JT, Orloff MM, Somaiah N, Tine BAV, Drabick JJ, et al. Newly updated activity results of alrizomadlin (APG-115), a novel MDM2/p53 inhibitor, plus pembrolizumab: Phase 2 study in adults and children with various solid tumors. J Clin Oncol. 2022;40:9517–9517. 10.1200/JCO.2022.40.16_suppl.9517.

[95] Ziogas, D.C.; Theocharopoulos, C.; Lialios, P.P.; Foteinou, D.; Koumprentziotis, I.A.; Xynos, G.; Gogas, H. Beyond CTLA-4 and PD-1 Inhibition: Novel Immune Checkpoint Molecules for Melanoma Treatment. *Cancers (Basel)* **2023**, *15*, 10.3390/cancers15102718. 10.3390/cancers15102718 PMC10216291 37345056

[96] Bol KF, Mensink HW, Aarntzen EH, Schreibelt G, Keunen JE, Coulie PG, de Klein A, Punt CJ, Paridaens D, Figdor CG, et al. Long overall survival after dendritic cell vaccination in metastatic uveal melanoma patients. Am J Ophthalmol. 2014;158:939–47. 10.1016/j.ajo.2014.07.014. 25038326 10.1016/j.ajo.2014.07.014

[97] Koch EAT, Schaft N, Kummer M, Berking C, Schuler G, Hasumi K, Dorrie J, Schuler-Thurner B. A One-Armed Phase I Dose Escalation Trial Design: Personalized Vaccination with IKKbeta-Matured, RNA-Loaded Dendritic Cells for Metastatic Uveal Melanoma. Front Immunol. 2022;13. 10.3389/fimmu.2022.785231. 35185883 10.3389/fimmu.2022.785231 PMC8854646

[98] Lin D, Shen Y, Liang T. Oncolytic virotherapy: basic principles, recent advances and future directions. Signal Transduct Target Ther. 2023;8:156. 10.1038/s41392-023-01407-6. 37041165 10.1038/s41392-023-01407-6 PMC10090134

[99] Sacco JJ, Harrington KJ, Olsson-Brown A, Chan TY, Nenclares P, Leslie I, Bommareddy P, Kalbasi A, Xie B, Mishal M, et al. Safety, efficacy, and biomarker results from an open-label, multicenter, phase 1 study of RP2 alone or combined with nivolumab in a cohort of patients with uveal melanoma. J Clin Oncol. 2024;42:9511–9511. 10.1200/JCO.2024.42.16_suppl.9511.

[100] Smith KER, Peng KW, Pulido JS, Weisbrod AJ, Strand CA, Allred JB, Newsom AN, Zhang L, Packiriswamy N, Kottke T, et al. A phase I oncolytic virus trial with vesicular stomatitis virus expressing human interferon beta and tyrosinase related protein 1 administered intratumorally and intravenously in uveal melanoma: safety, efficacy, and T cell responses. Front Immunol. 2023;14:1279387. 10.3389/fimmu.2023.1279387. 38022659 10.3389/fimmu.2023.1279387 PMC10644866

[101] Lutzky J, Sullivan RJ, Cohen JV, Ren Y, Li A, Haq R. Phase 1b study of intravenous coxsackievirus A21 (V937) and ipilimumab for patients with metastatic uveal melanoma. J Cancer Res Clin Oncol. 2023;149:6059–66. 10.1007/s00432-022-04510-3. 36651961 10.1007/s00432-022-04510-3 PMC10356892

[102] Garcia M, Moreno R, Gil-Martin M, Cascallo M, de Olza MO, Cuadra C, Piulats JM, Navarro V, Domenech M, Alemany R, et al. A Phase 1 Trial of Oncolytic Adenovirus ICOVIR-5 Administered Intravenously to Cutaneous and Uveal Melanoma Patients. Hum Gene Ther. 2019;30:352–64. 10.1089/hum.2018.107. 30234393 10.1089/hum.2018.107

[103] Chandran SS, Somerville RPT, Yang JC, Sherry RM, Klebanoff CA, Goff SL, Wunderlich JR, Danforth DN, Zlott D, Paria BC, et al. Treatment of metastatic uveal melanoma with adoptive transfer of tumour-infiltrating lymphocytes: a single-centre, two-stage, single-arm, phase 2 study. Lancet Oncol. 2017;18:792–802. 10.1016/S1470-2045(17)30251-6. 28395880 10.1016/S1470-2045(17)30251-6 PMC5490083

[104] Patel SP, Forget M-A, Kreidieh FY, Pelster M, Davies MA, Amaria RN, Gombos DS, Bernatchez C. Tumor infiltrating lymphocyte (TIL) harvest and ex vivo expansion from primary and metastatic (met) uveal melanoma (UM) tumors. J Clin Oncol. 2023;41:9513–9513. 10.1200/JCO.2023.41.16_suppl.9513.

[105] Hassel JC, Piperno-Neumann S, Rutkowski P, Baurain JF, Schlaak M, Butler MO, Sullivan RJ, Dummer R, Kirkwood JM, Orloff M, et al. Three-Year Overall Survival with Tebentafusp in Metastatic Uveal Melanoma. N Engl J Med. 2023;389:2256–66. 10.1056/NEJMoa2304753. 37870955 10.1056/NEJMoa2304753 PMC11188986

[106] Dimitriou F, Orloff MM, Koch Hein EC, Cheng PF, Hughes IF, Simeone E, Montazeri K, Grover P, Mehmi I, Gerard CL, et al. Treatment sequence with tebentafusp and immune checkpoint inhibitors in patients with metastatic uveal melanoma and metastatic GNA11/GNAQ mutant melanocytic tumors. Eur J Cancer. 2025;214. 10.1016/j.ejca.2024.115161. 39647344 10.1016/j.ejca.2024.115161

[107] Tomsitz, D.; Ruf, T.; Heppt, M.; Staeger, R.; Ramelyte, E.; Dummer, R.; Garzarolli, M.; Meier, F.; Meier, E.; Richly, H.; et al. Tebentafusp in Patients with Metastatic Uveal Melanoma: A Real-Life Retrospective Multicenter Study. *Cancers (Basel)* **2023**, *15*, 10.3390/cancers15133430. 10.3390/cancers15133430 PMC10341126 37444540

[108] Koch, E.C.; Arteaga Ceballos, D.P.; Vilbert, M.; Lajkosz, K.; Pimentel Muniz, T.; Hirsch, I.; Silva Almeida Ribeiro, M.F.; Mantle, L.; Anczurowski, M.; Hogg, D.; et al. 831P Outcomes of immune checkpoint inhibitors in patients with metastatic uveal melanoma treated with tebentafusp. *Annals of Oncology* **2022**, *33*, S928, 10.1016/j.annonc.2022.07.957.

[109] Zuidervaart W, van Nieuwpoort F, Stark M, Dijkman R, Packer L, Borgstein AM, Pavey S, van der Velden P, Out C, Jager MJ, et al. Activation of the MAPK pathway is a common event in uveal melanomas although it rarely occurs through mutation of BRAF or RAS. Br J Cancer. 2005;92:2032–8. 10.1038/sj.bjc.6602598. 15928660 10.1038/sj.bjc.6602598 PMC2361800

[110] Rimoldi D, Salvi S, Lienard D, Lejeune FJ, Speiser D, Zografos L, Cerottini JC. Lack of BRAF mutations in uveal melanoma. Cancer Res. 2003;63:5712–5. 14522889

[111] Khalili JS, Yu X, Wang J, Hayes BC, Davies MA, Lizee G, Esmaeli B, Woodman SE. Combination small molecule MEK and PI3K inhibition enhances uveal melanoma cell death in a mutant GNAQ- and GNA11-dependent manner. Clin Cancer Res. 2012;18:4345–55. 10.1158/1078-0432.CCR-11-3227. 22733540 10.1158/1078-0432.CCR-11-3227 PMC3935730

[112] Ambrosini G, Pratilas CA, Qin LX, Tadi M, Surriga O, Carvajal RD, Schwartz GK. Identification of unique MEK-dependent genes in GNAQ mutant uveal melanoma involved in cell growth, tumor cell invasion, and MEK resistance. Clin Cancer Res. 2012;18:3552–61. 10.1158/1078-0432.CCR-11-3086. 22550165 10.1158/1078-0432.CCR-11-3086 PMC3433236

[113] Carvajal RD, Sosman JA, Quevedo JF, Milhem MM, Joshua AM, Kudchadkar RR, Linette GP, Gajewski TF, Lutzky J, Lawson DH, et al. Effect of selumetinib vs chemotherapy on progression-free survival in uveal melanoma: a randomized clinical trial. JAMA. 2014;311:2397–405. 10.1001/jama.2014.6096. 24938562 10.1001/jama.2014.6096 PMC4249701

[114] Sacco JJ, Jackson R, Corrie P, Danson S, Evans TRJ, Ochsenreither S, Kumar S, Goodman A, Larkin J, Karydis I, et al. A three-arm randomised phase II study of the MEK inhibitor selumetinib alone or in combination with paclitaxel in metastatic uveal melanoma. Eur J Cancer. 2024;202. 10.1016/j.ejca.2024.114009. 38547774 10.1016/j.ejca.2024.114009

[115] Faiao-Flores F, Emmons MF, Durante MA, Kinose F, Saha B, Fang B, Koomen JM, Chellappan SP, Maria-Engler SS, Rix U, et al. HDAC Inhibition Enhances the In Vivo Efficacy of MEK Inhibitor Therapy in Uveal Melanoma. Clin Cancer Res. 2019;25:5686–701. 10.1158/1078-0432.CCR-18-3382. 31227503 10.1158/1078-0432.CCR-18-3382 PMC6744978

[116] Buchbinder EI, Cohen JV, Tarantino G, Lian CG, Liu D, Haq R, Hodi FS, Lawrence DP, Giobbie-Hurder A, Knoerzer D, et al. A Phase II Study of ERK Inhibition by Ulixertinib (BVD-523) in Metastatic Uveal Melanoma. Cancer Res Commun. 2024;4:1321–7. 10.1158/2767-9764.CRC-24-0036. 38683104 10.1158/2767-9764.CRC-24-0036 PMC11107576

[117] Wu X, Zhu M, Fletcher JA, Giobbie-Hurder A, Hodi FS. The protein kinase C inhibitor enzastaurin exhibits antitumor activity against uveal melanoma. PLoS ONE. 2012;7. 10.1371/journal.pone.0029622. 22253748 10.1371/journal.pone.0029622 PMC3257235

[118] Park JJ, Hamad SA, Stewart A, Carlino MS, Lim SY, Rizos H. PKC-independent PI3K signalling diminishes PKC inhibitor sensitivity in uveal melanoma. Oncogenesis. 2024;13:9. 10.1038/s41389-024-00511-8. 38418838 10.1038/s41389-024-00511-8 PMC10902289

[119] Piperno-Neumann S, Carlino MS, Boni V, Loirat D, Speetjens FM, Park JJ, Calvo E, Carvajal RD, Nyakas M, Gonzalez-Maffe J, et al. A phase I trial of LXS196, a protein kinase C (PKC) inhibitor, for metastatic uveal melanoma. Br J Cancer. 2023;128:1040–51. 10.1038/s41416-022-02133-6. 36624219 10.1038/s41416-022-02133-6 PMC10006169

[120] McKean M, Chmielowski B, Butler MO, Carvajal R, Rodon J, Carlino M, Kim KB, Wise-draper T, Khan S, Salama AKS, et al. 1081O ctDNA reduction and clinical efficacy of the darovasertib + crizotinib (daro + crizo) combination in metastatic uveal melanoma (MUM). Ann Oncol. 2023;34:S651. 10.1016/j.annonc.2023.09.2215.

[121] Joshua, A.M.; O'day, R.; Glasson, W.; Sia, D.; McGrath, L.; Ameratunga, M.; Cosman, R.; Cherepanoff, S.; O'Quigley, M.; Beaupre, D.M.; et al. A phase 2 safety and efficacy study of neoadjuvant/adjuvant darovasertib for localized ocular melanoma. *Journal of Clinical Oncology* **2024**, *42*, 9510–9510, 10.1200/JCO.2024.42.16_suppl.9510.

[122] Giacomo AMD, Simonelli M, Santangelo F, Amato G, Simonetti E, Graham J, Lahn MMF, Conza GD, Hammett T, Zorrilla R, et al. Roginolisib, an oral, highly selective and allosteric modulator of phosphoinositide 3-kinase inhibitor delta (PI3Kδ) in patients with uveal melanoma and advanced cancers. J Clin Oncol. 2024;42:9597–9597. 10.1200/JCO.2024.42.16_suppl.9597.

[123] Yu FX, Luo J, Mo JS, Liu G, Kim YC, Meng Z, Zhao L, Peyman G, Ouyang H, Jiang W, et al. Mutant Gq/11 promote uveal melanoma tumorigenesis by activating YAP. Cancer Cell. 2014;25:822–30. 10.1016/j.ccr.2014.04.017. 24882516 10.1016/j.ccr.2014.04.017 PMC4075337

[124] Feng, X.; Arang, N.; Rigiracciolo, D.C.; Lee, J.S.; Yeerna, H.; Wang, Z.; Lubrano, S.; Kishore, A.; Pachter, J.A.; Konig, G.M.; et al. A Platform of Synthetic Lethal Gene Interaction Networks Reveals that the GNAQ Uveal Melanoma Oncogene Controls the Hippo Pathway through FAK. *Cancer Cell* **2019**, *35*, 457–472 e455, 10.1016/j.ccell.2019.01.009. 10.1016/j.ccell.2019.01.009 PMC6737937 30773340

[125] Paradis JS, Acosta M, Saddawi-Konefka R, Kishore A, Gomes F, Arang N, Tiago M, Coma S, Lubrano S, Wu X, et al. Synthetic Lethal Screens Reveal Cotargeting FAK and MEK as a Multimodal Precision Therapy for GNAQ-Driven Uveal Melanoma. Clin Cancer Res. 2021;27:3190–200. 10.1158/1078-0432.CCR-20-3363. 33568347 10.1158/1078-0432.CCR-20-3363 PMC8895627

[126] Seedor, R.S.; Terai, M.; Majeed, A.; Tanaka, R.; Aplin, A.E.; Orloff, M.; Molinolo, A.A.; Gutkind, J.S.; Sato, T. Abstract CT260: A phase II trial of defactinib combined with avutometinib in patients with metastatic uveal melanoma. *Cancer Research* **2024**, *84*, CT260-CT260, 10.1158/1538-7445.Am2024-ct260. 10.3390/cancers18142232 PMC13407007 42512298

[127] Sorrentino G, Ruggeri N, Specchia V, Cordenonsi M, Mano M, Dupont S, Manfrin A, Ingallina E, Sommaggio R, Piazza S, et al. Metabolic control of YAP and TAZ by the mevalonate pathway. Nat Cell Biol. 2014;16:357–66. 10.1038/ncb2936. 24658687 10.1038/ncb2936

[128] Amaro, A.A.; Gangemi, R.; Emionite, L.; Castagnola, P.; Filaci, G.; Jager, M.J.; Tanda, E.T.; Spagnolo, F.; Mascherini, M.; Pfeffer, U.; et al. Cerivastatin Synergizes with Trametinib and Enhances Its Efficacy in the Therapy of Uveal Melanoma. *Cancers (Basel)* **2023**, *15*, 10.3390/cancers15030886. 10.3390/cancers15030886 PMC9913575 36765842

[129] Tanaka, R.; Terai, M.; Londin, E.; Sato, T. The Role of HGF/MET Signaling in Metastatic Uveal Melanoma. *Cancers (Basel)* **2021**, *13*, 10.3390/cancers13215457. 10.3390/cancers13215457 PMC8582360 34771620

[130] Khan S, Lutzky J, Shoushtari AN, Jeter J, Marr B, Olencki TE, Cebulla CM, Abdel-Rahman M, Harbour JW, Sender N, et al. Adjuvant crizotinib in high-risk uveal melanoma following definitive therapy. Front Oncol. 2022;12. 10.3389/fonc.2022.976837. 36106113 10.3389/fonc.2022.976837 PMC9465386

[131] Ohara, M.; Saito, K.; Kageyama, K.; Terai, M.; Cheng, H.; Aplin, A.E.; Sato, T. Dual Targeting of CDK4/6 and cMET in Metastatic Uveal Melanoma. *Cancers (Basel)* **2021**, *13*, 10.3390/cancers13051104. 10.3390/cancers13051104 PMC7961994 33806615

[132] He AR, Cohen RB, Denlinger CS, Sama A, Birnbaum A, Hwang J, Sato T, Lewis N, Mynderse M, Niland M, et al. First-in-Human Phase I Study of Merestinib, an Oral Multikinase Inhibitor, in Patients with Advanced Cancer. Oncologist. 2019;24:e930–42. 10.1634/theoncologist.2018-0411. 30833489 10.1634/theoncologist.2018-0411 PMC6738318

[133] Wallander ML, Layfield LJ, Emerson LL, Mamalis N, Davis D, Tripp SR, Holden JA. KIT mutations in ocular melanoma: frequency and anatomic distribution. Mod Pathol. 2011;24:1031–5. 10.1038/modpathol.2011.57. 21478825 10.1038/modpathol.2011.57

[134] Nathan PD, Marshall E, Smith CT, Bickerstaff M, Escriu C, Marples M, Damato B, Kalirai H, Coupland S. A Cancer Research UK two-stage multicenter phase II study of imatinib in the treatment of patients with c-kit positive metastatic uveal melanoma (ITEM). J Clin Oncol. 2012;30:8523–8523. 10.1200/jco.2012.30.15_suppl.8523.

[135] Hofmann UB, Kauczok-Vetter CS, Houben R, Becker JC. Overexpression of the KIT/SCF in uveal melanoma does not translate into clinical efficacy of imatinib mesylate. Clin Cancer Res. 2009;15:324–9. 10.1158/1078-0432.CCR-08-2243. 19118061 10.1158/1078-0432.CCR-08-2243

[136] Penel N, Delcambre C, Durando X, Clisant S, Hebbar M, Negrier S, Fournier C, Isambert N, Mascarelli F, Mouriaux F. O-Mel-Inib: a Cancero-pole Nord-Ouest multicenter phase II trial of high-dose imatinib mesylate in metastatic uveal melanoma. Invest New Drugs. 2008;26:561–5. 10.1007/s10637-008-9143-2. 18551246 10.1007/s10637-008-9143-2

[137] Landreville S, Agapova OA, Matatall KA, Kneass ZT, Onken MD, Lee RS, Bowcock AM, Harbour JW. Histone deacetylase inhibitors induce growth arrest and differentiation in uveal melanoma. Clin Cancer Res. 2012;18:408–16. 10.1158/1078-0432.CCR-11-0946. 22038994 10.1158/1078-0432.CCR-11-0946 PMC3261307

[138] Ny L, Jespersen H, Karlsson J, Alsen S, Filges S, All-Eriksson C, Andersson B, Carneiro A, Helgadottir H, Levin M, et al. The PEMDAC phase 2 study of pembrolizumab and entinostat in patients with metastatic uveal melanoma. Nat Commun. 2021;12:5155. 10.1038/s41467-021-25332-w. 34453044 10.1038/s41467-021-25332-w PMC8397717

[139] Grimes J, Shoushtari AN, Orloff M, Khan S, Chiuzan C, Hsiao SJ, McDonnell D, Marr BP, Carvajal RD. Clinical characteristics of SF3B1 mutant (mut) uveal melanoma (UM) and response to immune checkpoint inhibition (ICI). J Clin Oncol. 2021;39:9535–9535. 10.1200/JCO.2021.39.15_suppl.9535.

[140] Fong, J.Y.; Pignata, L.; Goy, P.A.; Kawabata, K.C.; Lee, S.C.; Koh, C.M.; Musiani, D.; Massignani, E.; Kotini, A.G.; Penson, A.; et al. Therapeutic Targeting of RNA Splicing Catalysis through Inhibition of Protein Arginine Methylation. *Cancer Cell* **2019**, *36*, 194–209 e199, 10.1016/j.ccell.2019.07.003. 10.1016/j.ccell.2019.07.003 PMC7194031 31408619

[141] Ito, K.; Thodima, V.; Carter, J.; Bhagwat, N.; Sivakumar, M.; Grego, A.; Rager, J.; Terai, M.; Sato, T.; Abdel-Wahab, O.; et al. *Abstract 1137: PRMT5 inhibition regulates alternative splicing and DNA damage repair pathways in SF3B1 R625G expressing uveal melanoma cells*; 2021; Volume 81, pp. 1137–1137.

[142] Cheung KL, Kim C, Zhou MM. The Functions of BET Proteins in Gene Transcription of Biology and Diseases. Front Mol Biosci. 2021;8. 10.3389/fmolb.2021.728777. 34540900 10.3389/fmolb.2021.728777 PMC8446420

[143] Segura MF, Fontanals-Cirera B, Gaziel-Sovran A, Guijarro MV, Hanniford D, Zhang G, Gonzalez-Gomez P, Morante M, Jubierre L, Zhang W, et al. BRD4 sustains melanoma proliferation and represents a new target for epigenetic therapy. Cancer Res. 2013;73:6264–76. 10.1158/0008-5472.CAN-13-0122-T. 23950209 10.1158/0008-5472.CAN-13-0122-T PMC4254777

